# Nickel Supplementation for Enhanced Soybean Growth: A Micrometric-to-Nanometric Investigation of Biological Nitrogen Fixation and Metabolism

**DOI:** 10.1021/acsomega.5c10937

**Published:** 2026-04-24

**Authors:** Jessica Bezerra de Oliveira, Antony van der Ent, Fernanda Viginotti Alves, Bruna Wurr Rodak, Nikolas de Souza Mateus, Nandhara Angelica Carvalho Mendes, Josué Martins Gonçalves, Koiti Araki, Hudson Wallace Pereira de Carvalho, André Rodrigues dos Reis, Fernando Shintate Galindo, José Lavres

**Affiliations:** † 28133 Universidade de São Paulo, Centro de Energia Nuclear Na Agricultura, Piracicaba, São Paulo 13400-970, Brazil; ‡ Wageningen University & Research, Laboratory of Genetics, Wageningen 6708 PB, The Netherlands; § 28108 Sao Paulo State University (UNESP), Tupã 17602496, Brazil; ∥ University of São Paulo, Institute of Chemistry, Department of Fundamental Chemistry, São Paulo, São Paulo 05508-000, Brazil; ⊥ Sao Paulo State University (UNESP), Dracena 17602496, Brazil

## Abstract

Nickel (Ni) plays a central role in plant nitrogen (N) metabolism, yet the long-term physiological impact of nanostructured Ni fertilizers on soybean (*Glycine max* L.) remains insufficiently characterized. This study assessed the effects of Ni seed treatment and foliar application, using macro-, micro-, and nanosized Ni sources, on soybean cv. “IPRO Flecha” N metabolism and biological nitrogen fixation (BNF) throughout the crop life cycle. Ni was applied as seed coating (45 mg Ni kg^–1^ seed) and/or foliar spray (20 g Ni ha^–1^ at V4) using NiSO_4_·6H_2_O (macro), micrometric Ni­(OH)_2_ (∼24 μm), or nanometric Ni­(OH)_2_ (∼5 nm). Key physiological, enzymatic, and isotopic parameters were measured at V6, R2, and R7 stages, including nitrogenase, urease, and nitrate reductase activities, BNF contribution (δ^15^N), and N metabolites (N-NH_3_, N-NO_3_^–^, N-ureides). At R2, nano-Ni seed + foliar increased leaf dry weight by 28% compared with seed-only and 49% compared with the control, whereas at R5 the same treatment reduced leaf dry weight by 62% relative to nano-Ni seed-only, indicating a strong stage-dependent response. At maturity (R7), Ni application increased seed yield across sources, with mean yield rising from 12 g pot^–1^ in the control to 19–22 g pot^–1^, with the highest yield under nano-Ni (22 g pot^–1^). Ni supply increased urease and nitrate reductase activities relative to the control; however, nano-Ni reduced nitrate reductase at V6 (≈41–43% lower than macro- and micro-Ni), and nano-Ni seed-only reduced nitrogenase activity at R2 by ∼33% compared with the control. BNF in shoots ranged from 60.5–87.4% at R2 and was generally higher under Ni treatments at R7 (≈85–92%), except for nano-Ni seed + foliar (67.3%). Overall, Ni improved soybean N metabolism and yield, but nano-Ni effects were highly dependent on the growth stage and application method, highlighting the need for mechanistic studies on nanoparticle behavior over time to support consistent agronomic outcomes.

## Graphical abstract

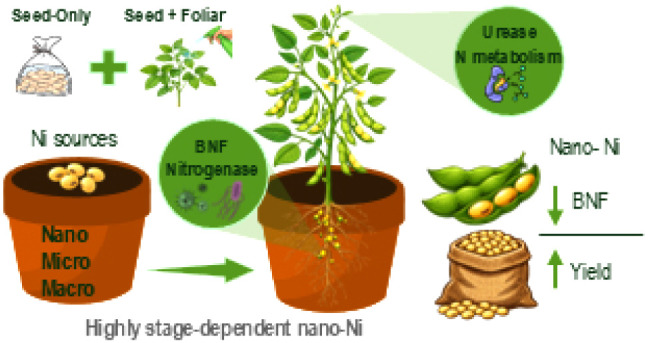

## 1 Introduction

Soybean (*Glycine max* *)* ranks among the major global crops and serves as a primary source of plant protein and edible oil.^1^ Enhancements in soybean yield have largely resulted from increases in biomass production and improved seed partitioning, both of which depend on substantial nitrogen (N) inputs.^2^ These inputs are primarily derived from biological nitrogen fixation (BNF) or soil-based N application.^3^ The contribution of BNF to soybean’s N requirements ranges from 0% to 98% and is influenced by several factors, with rhizobial activity being the most critical.^4^ BNF is facilitated by symbiotic interactions between soybean roots and bacteria of the *Bradyrhizobium* genus, which efficiently fix atmospheric nitrogen.^5^ Through root infection, these bacteria form nodules wherein atmospheric N_2_ is enzymatically reduced to ammonia (NH_3_).^6,7^ This biological pathway provides an efficient substitute for synthetic nitrogen fertilizers by lowering reliance on chemical inputs, improving plant nutritional status, and reducing environmental risks linked to excessive fertilization.^8,9^

Nitrogen is the nutrient most required by soybean plants, playing a critical role in their growth and productivity.^10^ To meet this substantial N demand, optimizing BNF is essential; however, the efficiency of BNF can be significantly hindered by the low availability of certain micronutrients, such as copper (Cu), iron (Fe), manganese (Mn), molybdenum (Mo), and nickel (Ni), as well as the low availability of the beneficial element cobalt (Co).^11^ This issue is particularly pronounced in tropical oxisols, where excessive lime applications often lead to micronutrient deficiencies, resulting in impaired nitrogen fixation capacity.^12^ Nickel is a vital micronutrient for plants, serving as a cofactor for several metalloenzymes, including urease and glyoxalase I, which are critical for N metabolism and stress responses.^13–16^ Additionally, Ni is essential for the expression and activity of hydrogenase, an enzyme that facilitates the BNF process by eliminating free hydrogen radicals (H^+^) generated during the reduction of atmospheric N_2_ to ammonia (NH_3_).^7,17^ Thus, adequate Ni availability is pivotal for maintaining efficient BNF and supporting the N economy of soybean plants.^18^

Despite its essential role in plant N metabolism, Ni has historically been overlooked in plant nutrition research and fertilizer management. For many years, Ni was not included in standard lists of essential micronutrients, largely because of its low quantitative requirement and the assumption that trace contamination from soils, fertilizers, or irrigation water was sufficient to meet plant demand. As a consequence, Ni has frequently been omitted from the formulation of nutrient solutions used in hydroponic and controlled-environment studies, where background Ni inputs are minimal or absent. This omission has contributed to the underestimation of Ni deficiency risks and to a limited understanding of its physiological relevance, particularly in crops with high reliance on BNF, such as soybean.^13,19^

The fulfillment of plants’ mineral nutrition requirements—whether through soil uptake, foliar spraying, or seed coating—is fundamental to plants’ growth and development.^20^ A deeper understanding of the Ni supply via seed treatment or foliar application and its subsequent impact on BNF is, therefore, crucial. Conventional Ni-based fertilizer, as well as micronutrients, can be supplied through soil amendments, seed treatments, or foliar sprays.^21^ Among these methods, seed treatment with micronutrients offers several benefits, including enhanced BNF, improved germination synchrony,^22,23^ reduced imbibition time,^24^ and the accumulation of germination-enhancing metabolites.^25,26^ While any of these approaches can meet a crop’s micronutrient demands, foliar application has proven particularly effective in improving yields and enriching seed quality.^27^

Foliar nutrient application is widely adopted as a strategy to alleviate nutrient deficiencies^28,29^ or in conjunction with soil fertilization to optimize nutrient use while reducing annual fertilizer inputs.^21^ This approach has been effective for crops such as soybean, rice, and maize, mitigating nutrient toxicity symptoms.^27,30–32^ Foliar sprays are highly efficient, minimizing the lag time between nutrient application and plant uptake.^33,34^ However, the effectiveness of foliar fertilization depends on the plant species, the plant’s nutritional status, and the chemical properties of the fertilizer.^35–37^ In recent years, researchers have increasingly investigated the capacity of nanofertilizers to improve crop productivity.^38–40^ Nanofertilizers offer unique advantages due to their controlled release properties and increased bioavailability, providing an innovative approach to improving nutrient efficiency and crop performance.^41,42^ However, research in this area remains limited, particularly regarding its application in addressing micronutrient deficiencies in key crops like soybeans.

The integration of nanotechnology into agriculture is reshaping production systems through strategies aimed at increasing crop performance, optimizing nutrient efficiency, and supporting environmental sustainability.^43,44^ Recent investigations have drawn attention to nanoparticles functioning as sensors, herbicidal and fungicidal agents, and nutrient carriers, highlighting their potential to alleviate stresses such as salinity, abiotic constraints, and nutrient shortages.^41,45,46^ Nanofertilizers, in particular, stand out for their ability to deliver nutrients gradually and with greater precision than conventional fertilizers.^47,48^ This targeted delivery minimizes environmental risks, such as soil pollution, while reducing the quantities of fertilizers required.^49,50^ Applied as foliar treatments or through alternative delivery systems, nanofertilizers have proven effective for crop use by providing a controlled and sustained nutrient supply relative to traditional fertilizers.^39,43,51,52^ This is particularly true when the application rates of nanofertilizers are agronomically and environmentally realistic, ensuring efficient nutrient uptake without posing risks of overapplication or environmental harm.^46,53,54^ For instance, Fe nanoparticles have shown improved nutrient uptake and reduced oxidative stress, particularly in crops grown on contaminated soils.^55^ Similarly, Zn nanoparticles enhance the biosynthesis of photosynthetic pigments and boost plant biomass, especially under stress conditions.^56^ Moreover, the concept of nanopriming—treating seeds with nanoparticles—has demonstrated promising results in improving germination and seedling vigor.^30,35,57,58^ This method facilitates water uptake and biochemical activation, leading to better crop establishment.^59^ Despite these advancements, the potential applications of Ni nanoparticles remain relatively underexplored, particularly concerning their interactions with N metabolism and BNF in legumes like soybean. To date, only a few studies have examined the effects of Ni-based nanoparticles on soybean physiology following seed application^35^ or soil fertilization.^60^

Our hypothesis is that Ni application through seed treatment and foliar sprays, using different Ni sources and particle sizes, will interact synergistically in upregulating soybean growth, N metabolism, and BNF. Specifically, we suggest that (i) Ni nanoparticles (Ni-NPs) will enhance Ni bioavailability and uptake compared to micrometric or conventional Ni forms (*e.g.,* Ni sulfate); (ii) Ni-NPs will increase nitrate reductase and urease activity, enhancing N metabolism and the accumulation of key metabolites like ammonia (NH_3_), nitrate (NO_3_^−^), and ureides; (iii) soybean plants treated with Ni-NPs will show higher BNF efficiency due to enhanced nitrogenase functionality in root nodules; and that (iv) combining seed and foliar Ni-NP applications will maximize atmospheric N_2_ fixation, improving N contribution to plant growth and seed production relative to conventional Ni fertilizers applied solely via seed or foliar methods. In order to test these hypotheses, we undertook a multilevel approach at a range of nodule, leaf, and plant levels: (i) to assess plant growth under different Ni sources and application strategies; (ii) to measure urease, nitrate reductase, and nitrogenase enzyme activities; (iii) to investigate nitrogen metabolism by determining ammonia, nitrate, and ureide concentrations; and (iv) to estimate biological nitrogen fixation using the ^15^N natural abundance technique (δ^15^N ‰).

## 2 Materials and Methods

### 2.1 Soil Chemical Characterization and Conditions

The greenhouse trial was performed at the Department of Soil Science, University of São Paulo (USP), Piracicaba, Brazil. The experimental substrate corresponded to a Red-Yellow Latosol according to the Brazilian classification scheme,^61^ equivalent to an oxisol in soil taxonomy.^62^ Samples were taken from the upper 20 cm of soil in Itatinga, São Paulo State, Brazil (22°43′31″ S, 47°38′57″ W). The soil had a sandy texture and a low Ni concentration of 0.03 mg kg^–1^ as determined using the DTPA extraction method.^63^ The soil chemical compositions are as follows: pH (CaCl_2_) 4.2; organic matter (total carbon content) 5 g kg^–1^; available phosphorus (resin) 2 mg kg^–1^; exchangeable potassium (K) 0.3 mmol_a_ kg^–1^; exchangeable calcium (Ca) 1 mmol_a_ kg^–1^; exchangeable magnesium (Mg) 1 mmol_a_ kg^–1^; potential acidity (H+Al) 25 mmol_a_ kg^–1^; exchangeable aluminum (Al) 3 mmol_a_ kg^–1^; cation exchange capacity (CEC) 27 mmol_a_ kg^–1^; and exchangeable sulfur (S) 6 mg kg^–1^. Based on this analysis, soil correction was performed for each experimental unit to meet soybean nutritional requirements.^63^ Soil acidity was corrected to pH 5.5 by liming each pot individually according to the base saturation approach. The amendment consisted of a 3:1 blend of calcium carbonate (CaCO_3_) and magnesium carbonate (MgCO_3_), with application rates calculated separately for each treatment.

### 2.2 Soil Preparation and Fertilization

The soil used in this study had no history of legume cultivation; therefore, to ensure adequate initial N supply for all treatments, a low dose of ammonium nitrate (NH_4_NO_3_) was supplied at 166.75 mg N per pot (equivalent to 30 kg ha^–1^) to promote early plant establishment.^18,64,65^ Potassium fertilization consisted of KCl at 150 mg kg^–1^, incorporated at sowing, and reapplied as a top dressing during the V1 phenological stage (first leaflet emergence). Phosphorus was applied as calcium dihydrogen phosphate (CaH_2_PO_4_) at 300 mg kg^–1^. Micronutrients were added at the following rates per pot: 0.023 g of H_3_BO_3_, 0.038 g of FeSO_4_, 0.026 g of CuSO_4_, 0.056 g of MnSO_4_, and 0.087 g of ZnSO_4_, applied the day before planting.

### 2.3 Preparation of Nano Nickel Hydroxide – Ni­(OH)_2_

A suspension of stabilized α-nickel hydroxide nanoparticles was prepared following the sol–gel polyol method described previously.^66^ Briefly, a 1.0 mol L^–1^ KOH solution in n-butanol was added to a nickel acetate tetrahydrate solution in anhydrous glycerin (0.04 g mL^–1^) to achieve an OH^–^/Ni^2+^ molar ratio of 2:1. The reaction was stirred for 6 h and aged for 2 weeks prior to solvent removal, yielding a stable green α-Ni­(OH)_2_ nanoparticle suspension (∼5 nm) with no additional capping or functionalizing agents. Structural morphology and primary particle dimensions of α-nickel hydroxide nanoparticles were analyzed by transmission electron microscopy (TEM) and scanning electron microscopy (SEM).^66–68^

### 2.4 Experimental Setup

The study followed a completely randomized design comprising seven treatments organized in a 2 × 3 factorial arrangement (Figure 1). The first factor was the method of nickel (Ni) application: (1) seed-only (Seed) or (2) seed and foliar (Seed + Foliar at the V4 vegetative stage). The second factor was the Ni source: (1) NiSO_4_ (Macro), (2) Ni­(OH)_2_ (Micro), and (3) Ni­(OH)_2_ (Nano). For comparison with the nickel nanoparticle (Ni­(OH)_2_-NP) treatments, the conventional soluble Ni source (Nickel­(II) sulfate, NiSO_4_) is hereafter referred to as “macro-Ni”. Although NiSO_4_ is fully ionic in solution, the hydrated Ni^2+^ ion has an effective radius of ∼4.0 Å (0.40 nm), which is orders of magnitude smaller than the nanosized nickel hydroxide particles. The term “macro” is therefore used only as a practical label to distinguish the conventional, molecular-scale Ni source from the nanoparticle treatment. Each treatment included 11 replicates, distributed as follows: I) three replicates were harvested at the inflorescence emergence (R2 phenological stage); II) three replicates were harvested at the early seed development (R5 stage, ∼3 mm seeds) to evaluate root nodulation, urease activity, nitrate reductase activity, nitrogen content, and ureides; and III) the remaining five replicates were maintained until the fruit and maturity (R7 stage) for yield-related assessments. Each pot containing two soybean plants was considered to be one experimental unit. Seven treatments were evaluated: (1) control without Ni addition, with seeds treated only with Co and Mo and inoculated with *B. japonicum*; (2) NiSO_4_ (Macro–Seed), consisting of seed treatment with NiSO_4_·6H_2_O; (3) NiSO_4_ (Macro–Seed + Foliar), consisting of seed treatment plus foliar application at the V4 stage; (4) Ni­(OH)_2_ (Micro–Seed), with microscale Ni­(OH)_2_ applied via seed; (5) Ni­(OH)_2_ (Micro–Seed + Foliar), with microscale Ni­(OH)_2_ applied via seed and foliar fertilization; (6) Ni­(OH)_2_ (Nano–Seed), with nanoscale Ni­(OH)_2_ applied via seed; and (7) Ni­(OH)_2_ (Nano–Seed + Foliar), with nanoscale Ni­(OH)_2_ applied via seed and foliar fertilization. Preparation of the Ni hydroxide suspensions, including dispersion procedures and pH adjustment, is described by Oliveira et al. (20022b).^28^

**Figure 1:**
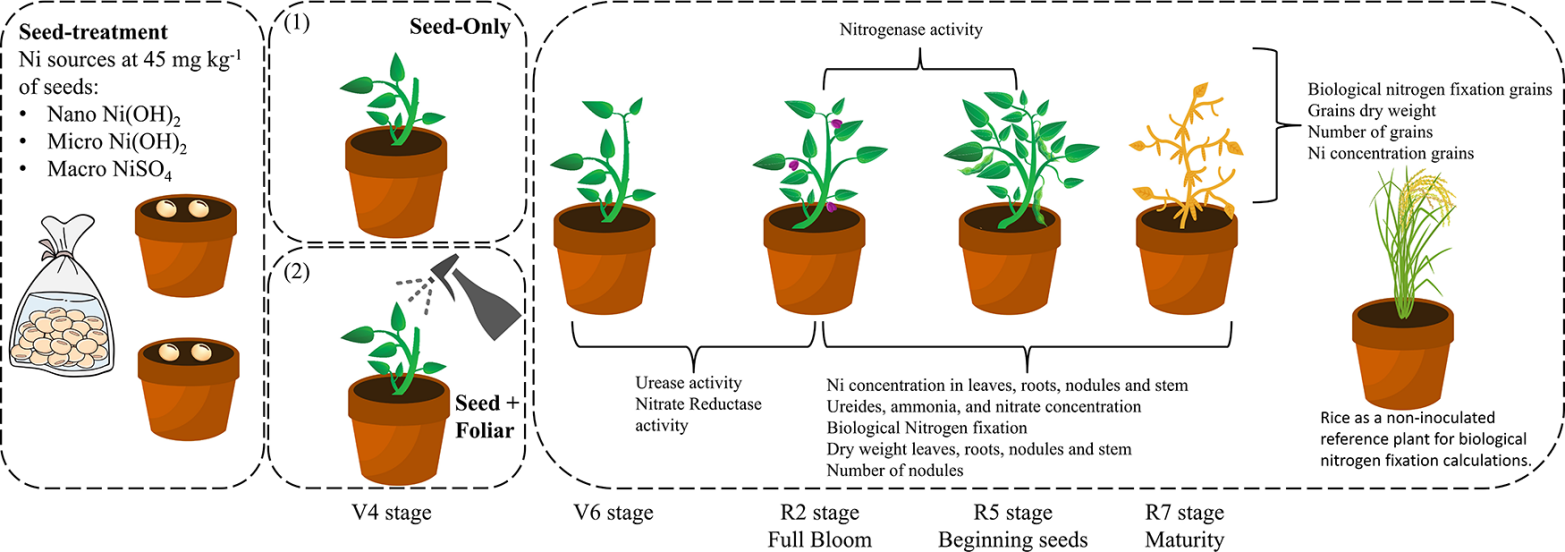
Illustration of the experimental design. Various parameters were assessed at the following four soybean growth stages: V6 (vegetative stage), R2 (full bloom), R5 (beginning seed), and R7 (maturity). At the V4 stage, foliar Ni application was administered to the designated pots using different Ni sources. The experiment utilized a consistent application rate of 45 mg Ni kg^–1^ of seeds, with Ni supplied in three particle sizes: nano, micro, and macro.

### 2.5 Plant Material and Cultivation Assay

The experiment used soybean (*Glycine max* (L.) Merr.) cultivar 6266 RSF IPRO Flecha, a high-yielding genotype. The cultivar has an indeterminate growth pattern, matures early, and belongs to maturity group 6.6.^69^ The experiment commenced at the seedling emergence stage (VE), and plants were later collected at three growth stages: full flowering (R2), early seed formation (R5), and physiological maturity (R7), which marked the end of the reproductive phase. Plants were grown in plastic pots filled with 3 kg of soil. Prior to sowing, 1 kg of seeds was treated with an aqueous commercial formulation containing molybdenum (Mo) and cobalt (Co) at 0.17 μL, equivalent to 90 mL of ha^–1^. Nickel (Ni) was then applied to each seed source at 45 mg kg^–1^ of seeds, corresponding to 2.5 g of ha^–1^. The treatment solution was supplemented with 2 μL of the commercial inoculants Semia 5019 and Semia 5079, with inoculants based on *Bradyrhizobium elkanii* and *Bradyrhizobium japonicum*, respectively. Seed treatment was carried out during the morning, followed by sowing at a 2.0 cm depth. Initially, 10 seeds were placed in each pot; at the V1 growth stage, seedlings were thinned to two plants per pot, and excess plants were discarded appropriately. No fungicides or insecticides were included in the seed treatment. The soybean plants were grown in a greenhouse for the duration of their growth cycle under a controlled 14 h light and 10 h dark photoperiod. Pots were irrigated using an automated irrigation system (Galcon, Kfar Blum, Israel). Prior to experiment initiation, soil moisture was adjusted to approximately 75% of the field capacity using tensiometers. Soil moisture was maintained near 70% of maximum water retention capacity during the experimental period by daily monitoring with tensiometers. Greenhouse air temperature was maintained at 27 °C during the day and 22 °C at night by using a computer-controlled environmental control system (Van der Hoeven, Monster, The Netherlands).

Rice (*Oryza sativa*), a non-atmospheric nitrogen-fixing species, was grown in the same soil and greenhouse conditions to serve as a reference crop for estimating soil^1,5^ N enrichment.^18,70–73^ Treatments included Ni applied either to seeds or as a foliar spray at the V4 vegetative stage,^74^ using aqueous preparations of different Ni sources. The application rate followed the recommendation of Barcelos et al.,^75^ equivalent to 20 g ha^–1^ of Ni. To minimize nutrient loss associated with the low solubility of hydroxide-based sources, we freshly prepared the spray solution and applied it individually to each plant. During application, pots were covered with aluminum foil to prevent contact between the solution and the soil surface, and care was taken to avoid runoff.

### 2.6 Concentrations of Nickel and Nutritional Analysis of Soybean Plants

At the R2 and R5 growth stages, harvested plants were partitioned into leaves, stems, nodules, and roots for subsequent determination of nitrogenase activity. Collected plant material was stored in labeled paper bags and dried at 65 °C for 72 h in a forced-air oven until a stable mass was obtained. Dry weight was recorded by using a precision balance. The dried samples were subsequently milled in a Wiley grinder and passed through a 1 mm sieve. The processed material was then forwarded for laboratory analysis to quantify macro- (excluding N) and micronutrient concentrations according to the method described by Lavres et al.^76^ Plant tissues were digested using a closed-vessel microwave system (TC Plus Labstation, Milestone, Sorisole, Italy) with a nitric acid (HNO_3_) and hydrogen peroxide (H_2_O_2_) mixture according to USEPA method 3051a.^77^ After digestion, solutions were transferred to 50 mL Falcon tubes and diluted to volume with Milli-Q water. Elemental concentrations were determined by inductively coupled plasma mass spectrometry (ICP-MS; Agilent 7500ce, Agilent Technologies, Tokyo, Japan). Method performance was verified using certified standard reference materials (NIST 1515, apple leaves; NIST 1573a, tomato leaves), with elemental recoveries ranging from 92% to 98%.

At the final harvest (R7 stage), plants were divided into seeds, nodules, roots, leaves, and stems for analysis of nutrient content, ureides, and natural ^15^N isotopic abundance (δ^15^N ‰). Earlier physiological assays were conducted without destructive sampling: urease and nitrate reductase activities were measured on the third or fourth fully expanded leaf from the apex of the main stem, including the petiole, at the V6 and R2 stages.

### 2.7 Evaluation of Total Nitrogen and Quantification of Biological Nitrogen Fixation through the Natural Abundance of ^15^N

The contribution of BNF was estimated using the natural abundance of ^15^N, following established isotopic procedures.^78,79^ This approach relies on the principle that soil nitrogen is typically slightly enriched in ^15^N relative to atmospheric N_2_. Consequently, nonfixing reference plants cultivated in the same soil display higher δ^15^N values, indicating their reliance on soil-derived nitrogen. In contrast, nitrogen-fixing plants incorporate atmospheric N_2_, resulting in lower δ^15^N signatures. As with other isotopic methods, the technique assumes that fixing and nonfixing plants access soil N with comparable ^15^N enrichment.^70,78,80^ To minimize bias, a species used as a reference standard, with root architecture and nitrogen demand similar to soybean, was selected. The fraction of plant nitrogen originating from atmospheric fixation was determined using the equation described by Shearer et al.^78^

$$\mathrm{\%BFN}=\mathrm{100 \times}\;(\delta^{15}{\mathrm{N reference}\;-\;\delta }^{15}\mathrm{N soybean})/\left(\delta^{15}\mathrm{N reference - B}\right)$$

In this equation, %BFN represents the proportion of N in soybean derived BNF. The term δ ^15^N reference corresponds to the natural abundance of^1,5^ N measured in the non–N-fixing reference plant, whereas δ ^15^N soybean indicates the natural ^15^N abundance detected in soybean tissue. The parameter B describes the isotopic fractionation factor, expressing the relative contribution of ^15^N compared with ^14^N during soybean N uptake from soil sources. A mean B value of −1.85‰, derived from the experimental data,^80^ was used for subsequent calculations. Oven-dried and ground subsamples were analyzed for total nitrogen (%N) and isotopic composition (δ^15^N) using an automated isotope ratio mass spectrometer coupled with an ANCA-GSL elemental analyzer (Sercon Co., UK). Total N content and the ^15^N/^14^N ratio were calculated following the procedure described by Barrie et al.^81^

### 2.8 Assessment of Urease Enzyme Activity

In vivo urease activity was assessed at the V6 and R2 stages following a modified version of the established procedure.^82^ Fresh plant samples were collected, sealed in plastic bags, and transported in insulated polystyrene containers to preserve enzyme integrity. Approximately 200 mg of green leaf tissue, cut into ∼1 mm segments, was incubated in 8 mL of NaH_2_PO_4_–urea buffer (pH 7.4) for 3 h at 30 °C. Samples were shielded from light with aluminum foil and maintained under continuous agitation during incubation.

After incubation, a 0.5 mL aliquot of the extract was transferred to a test tube and mixed with 2.5 mL of Reagent I (0.1 mol L^–1^ phenol with 50 mg of sodium nitroprusside) and 2.5 mL of Reagent II (0.125 mol L^–1^ NaOH, 0.15 mol L^–1^ Na_2_HPO_4_·12H_2_O, and 3% NaOCl). Tubes were sealed to prevent ammonia volatilization and incubated in a 37 °C water bath for 35 min. The resulting color development was quantified spectrophotometrically at 625 nm. Urease activity was calculated from the amount of NH_4_^+^ produced, using an NH_4_Cl calibration curve. Absorbance readings were obtained with a Cary 60 UV–Vis spectrophotometer (Agilent Technologies, Santa Clara, CA, USA).

### 2.9 Assessment of Nitrate Reductase Activity

In vivo nitrate reductase activity was measured at the V6 and R2 growth stages following a modified protocol based on a study by Mulder et al.^83^ Previously prepared plant samples were transferred to test tubes containing 4 mL of a KNO_3_ solution, which served as the nitrate source for the enzyme present in the leaf tissue. During the assay, nitrate reductase catalyzed the reduction of the concentration of NO_3_^–^ to NO_2_^–^. The tubes were incubated for 2 h at 37 °C in darkness by wrapping them in aluminum foil, with gentle mixing every 5 min to maintain uniform conditions. After incubation, a 1 mL aliquot of extract was collected, and the enzymatic reaction was terminated by adding 1 mL of sulfanilic acid. After a 5–10 min stabilization period, 1 mL of α-naphthylamine reagent was introduced and mixed thoroughly, followed by the addition of 1 mL of sodium acetate (C_2_H_3_NaO_2_). Absorbance was recorded at 540 nm. Nitrate reductase activity was quantified from nitrite production by using a calibration curve prepared with NaNO_2_ standards. Measurements were performed using a Cary 60 UV–Vis spectrophotometer (Agilent Technologies, Santa Clara, CA, USA).

### 2.10 Assessment of Nitrogenase Activity

Indirect quantification of nitrogenase activity (ANase) was performed through the acetylene reduction method.^18,70^ Plants were harvested at the R2 and R5 phenological stages, and the root systems were carefully removed from the soil to maintain intact nodules. The nodulated roots were immediately transferred to airtight flasks. Approximately 10% of the headspace gas (≈1 mL) was withdrawn from each flask by using a syringe and replaced with an equal volume of acetylene.

Because reported incubation times vary widely in the literature, a 1-h incubation period was adopted to minimize potential interference from stopper materials that may catalyze acetylene conversion. Following incubation, a 1 mL headspace gas sample was withdrawn using a 2.5 mL syringe and introduced into a gas chromatograph (Thermo Scientific Finnigan Trace GC 2000) fitted with dual Porapak N columns.^70^ Ethylene formation was measured and used as a proxy for nitrogenase activity.

Acetylene reduction values were initially expressed in parts per million (mg mL^–1^) and subsequently converted to micromoles per hour of incubation (μmol h^–1^) according to the equation below:

ANase=(V ×
UCG × At × F)/(i × h × number of plants)=UCG × 0.0430443

Where,

ANase = nitrogenase activity was expressed as mmol C_2_H_4_ h^–1^ plant^–1^ and calculated using the following parameters: flask volume (V) = 50 mL; chromatographic peak area (UCG) = area produced; attenuation factor (At) = 16; calibration constant (F) = 1.0761 × 10^–5^ mmol C_2_H_4_ per UCG unit; injected gas volume (i) = 1 mL; incubation time (h) = 1 h; and two plants per experimental unit.

### 2.11 Determination of Ammonia and Nitrate Concentration in Plant Tissue

Ammonia concentration was quantified in soybean stem tissue harvested at the R7 maturity stage. Approximately 1.0 g of fresh material was homogenized in 10 mL of extraction solvent containing 60% (v/v) methanol and 25% (v/v) chloroform. Following centrifugation at 13,200 rpm for 5 min, the clarified supernatant was collected for analysis. Determination of ammonium $\left(NH_{4}^{+}\right)$ followed method^84^ using a colorimetric assay. An aliquot of 150 μL of extract was reacted with 2.0 mL of reagent prepared by combining phenol and phosphate solutions in equal proportions. The phenol solution consisted of 2.5 g C_6_H_5_OH and 12.5 mg Na_2_Fe­(CN)_5_NO dissolved in 250 mL, while the phosphate solution contained 1.25 g NaOH, 13.4 g NaH_2_PO_4_, and 2.5 mL of 5% NaClO in 250 mL. Reaction mixtures were incubated at 37 °C for 1 h, and absorbance was measured at 630 nm to estimate ammonia concentration.

Nitrate content in R7 soybean stems was analyzed according to the method by Mulder et al.^83^ A 0.1 mL portion of the water-soluble MCW extract was treated with 0.4 mL of 5% (w/v) salicylic acid prepared in H_2_SO_4_ and allowed to react for 20 min at room temperature. Following this step, 9.5 mL of 2 N NaOH was gradually introduced. After the solution cooled to ambient temperature, the absorbance was read at 410 nm using a spectrophotometer. Nitrate concentrations were obtained by comparison with the sodium nitrate calibration curve.

### 2.12 Determination of Ureides

The ureide concentration of R7 soybean stems was quantified by calculating the total amounts of allantoin and allantoic acid present in the tissue. Measurements were performed according to the protocol outlined by Vogels and van Der Drift.^85^ The assay determination involved two consecutive hydrolysis reactions. During the alkaline phase, 250 μL of the MCW extract supernatant was combined with an equal volume of 0.5 M NaOH and a drop of C_6_H_8_N_2_. The mixture was heated at 100 °C for 8 min to convert allantoin into allantoic acid, then cooled to room temperature.

Acid hydrolysis was subsequently performed by adding 250 μL of 0.65 N HCl and reheating the solution at 100 °C for 4 min, promoting the conversion of allantoic acid to glyoxylate. After the mixture was cooled, 250 μL of 0.4 M phosphate buffer (pH 7.0) and 250 μL of 0.33% C_6_H_8_N_2_ were introduced, and the reaction was allowed to proceed for 5 min at room temperature before an additional 5-min incubation in an ice bath. The reaction was completed by adding 1.25 mL of prechilled concentrated HCl, followed by 250 μL of 1.65% C_6_FeK_4_N_6_, with vigorous vortex mixing. Following a 15-min incubation at room temperature, absorbance was recorded at 535 nm. Ureide concentration was quantified using an allantoin standard calibration curve.

### 2.13 Dry Matter Accumulation and Seed Yield

Plants were harvested at the R2, R5, and R7 growth stages and separated into leaves, stems, roots, and nodules. Each plant component was transferred to labeled paper bags, dried in a forced-air oven at 65 °C for 72 h, and subsequently weighed to determine the dry biomass using a precision balance. Seed yield was assessed independently by recording the mass of dried seeds.

### 2.14 Number of Nodules and Dry Weight

Nodule assessment was performed for each treatment and sampling stage by separating the nodules from the roots and recording their total number. After they were collected, nodules were oven-dried at 65 °C for 72 h, and their dry weight was measured.

### 2.15 Statistical Analysis

Statistical analyses were performed in the R environment (version 4.5.2; 2025). Analysis of variance (ANOVA) was conducted through F-tests within a mixed-effects modeling framework using the lme4 package in R,^86^ along with data manipulation facilitated by dplyr^87^ and tidyr.^88^ Model assumptions were verified by examining the residual normality with the Shapiro–Wilk test. When deviations from normality were detected, the affected variables were log_10_-transformed before fitting the final models. Post hoc comparisons, including Tukey’s test, were performed using the emmeans package.^89^ A significant level of 5% (*p* < 0.05) was applied to all analyses. Multivariate analyses, including hierarchical clustering and principal component analysis (PCA), were carried out with the factoextra package.^90,91^ Cluster patterns were visualized through heatmaps produced using pheatmap, and graphical outputs were created with the ggplot2 package.^92^

## 3 Results

### 3.1 Development of Soybean Plants and Biomass Yield

Overall, the dry weight varied considerably among treatments over time and across plant tissues. Some treatments promoted growth at certain stages, while others reduced it.

At R2, the application of nano-Ni seed + foliar significantly increased leaf dry weight by 28% compared to seed-only and 49% compared to the control (Figure 2a). Leaf dry weight ranged from 8.65 g in micro-Ni to 20.51 g in nano-Ni (seed-only) treatments. A comparable trend was observed in stems and roots, where seed-applied nano-Ni increased dry mass by 31% and 56%, respectively, relative to the micro-Ni treatment. At R5, contrasting effects were observed. The seed + foliar nano-Ni treatment reduced leaf dry weight by 62% compared to seed-only nano-Ni (Figure 2b). However, seed-only nano-Ni increased leaf dry weight by 24% relative to micro-Ni, ranging from 19 g (micro-Ni) to 33.5 g (nano-Ni). Seed-only nano-Ni also significantly outperformed both macro- and micro-Ni sources at this stage.

**Figure 2:**
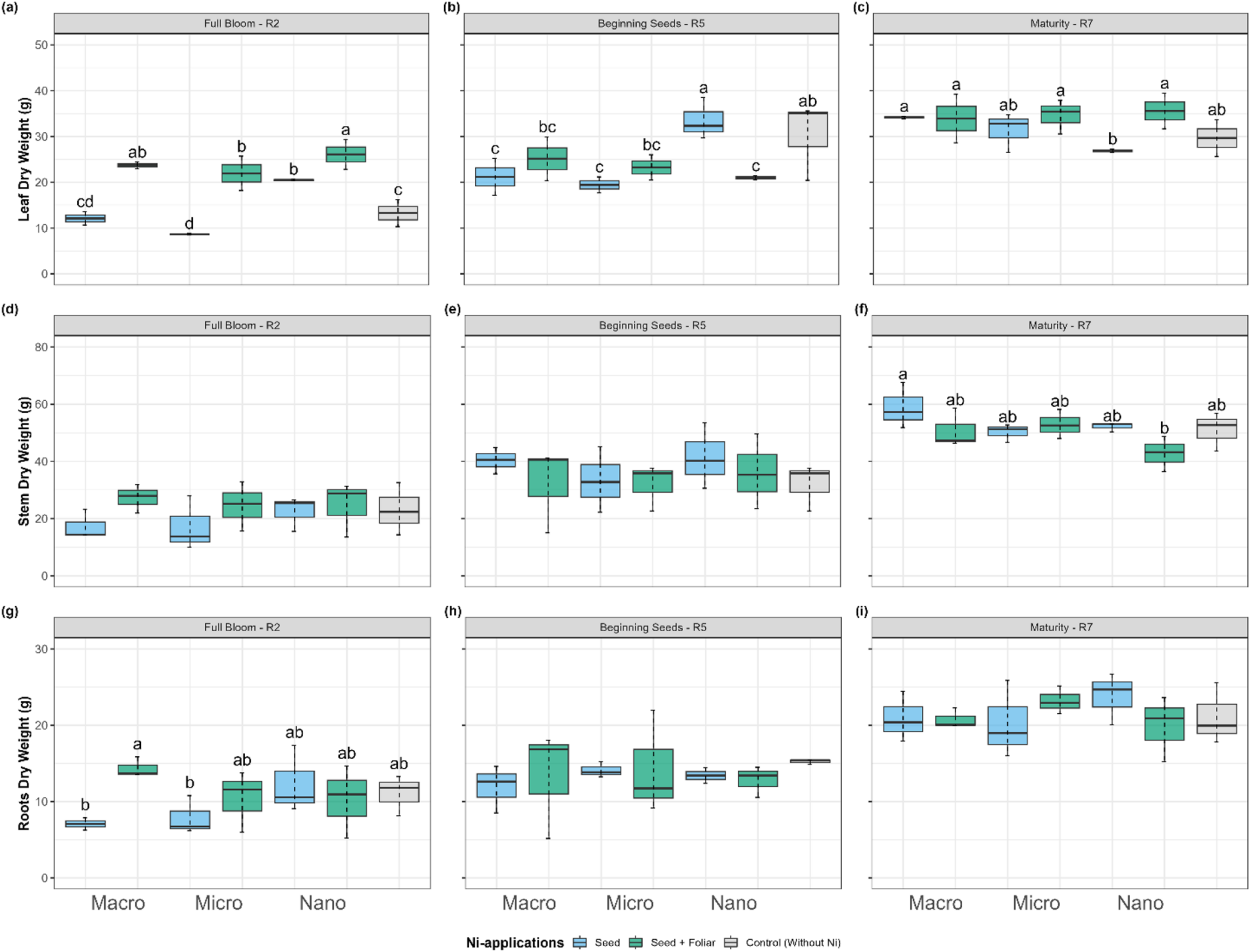
Effect on (a-*b*-c) leaf, (d-e-f) stem, and (g-h-i) root dry weight in soybean at (a-d-g) flowering (R2), (b-e-h) beginning seeds (R5), and (c-f-i) maturity (R7) of plants treated with Ni sources—macro, micro, and nanoparticles—applied via seed and foliar. Values are means and standard deviations of three (R2 and R5) and five (R7) replicates. Different letters indicate significant differences according to the least difference (Tukey) test, *P* < 0.05. Combined letters (e.g., “bc”) indicate overlap between statistical groups.

At R7, most treatments did not differ statistically, except for the difference between seed-only macro-Ni and nano-Ni (Figure 2c). In general, seed + foliar nano-Ni showed reduced dry weight in stems and roots at this stage. Still, seed-only nano-Ni outperformed macro-Ni, producing 69% more leaf dry matter, 29% more stem dry matter, and 74% more root dry matter at R2. At later stages, however, especially R7, the macro-Ni seed-only treatment surpassed nano-Ni in leaf and stem dry weight.

When yield was evaluated at R7 (fruiting/maturity stage), Ni application, regardless of source or method, significantly increased seed yield compared to the control (Figure 3). The highest yield was recorded in nano-Ni, with an average of 22 g per pot, followed by micro-Ni (21 g), macro-Ni (19 g), and the control (12 g). Relative to the control, dry weight increases were 86%, 83%, and 65% for nano-, micro-, and macro-Ni, respectively.

**Figure 3:**
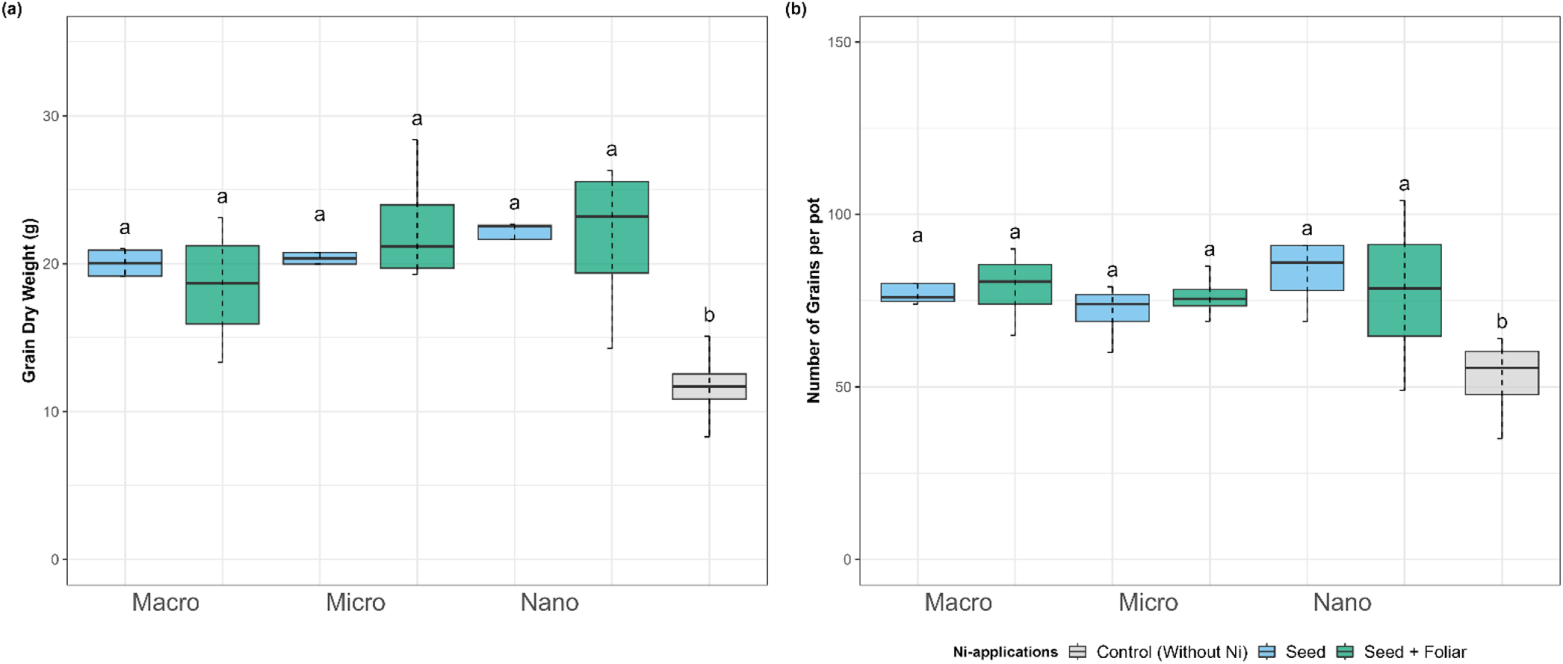
Effect on (a) dry weight and (b) number of grains at maturity (R7) of soybean plants treated with Ni sources—macro, micro, and nanoparticles—applied via seed and foliar. Values are means and standard deviations of five replicates. Different letters indicate significant differences according to the least difference (Tukey) test, *P* < 0.05. Combined letters (e.g., “bc”) indicate overlap between statistical groups.

Seed counts followed a similar pattern. Nano-Ni produced the highest number of seeds (80 per pot), closely followed by micro-Ni (74) and macro-Ni (79). In contrast, the control produced only 52 seeds per pot.

### 3.2 Nickel Concentrations in Soybean Tissues

Nickel concentrations in soybean tissues increased significantly in both Ni application methods (seed-only and seed + foliar) relative to those in the control (Table 1). At the R2 phenological stage, Ni concentrations in leaves increased by 1.39- and 1.73-fold in the nano- and macro-Ni, respectively, in the seed + foliar treatments relative to the control. In the seed-only treatment, Ni concentrations in leaves increased by 1.9-, 1.4-, and 1.7-fold in the micro-, nano-, and macro-Ni treatments, respectively, in comparison with the untreated control (no Ni application). At R2, Ni concentration in nodules increased by 1.38- and 1.42-fold with nano- and macro-Ni seed-only treatments compared to the control; however, for the other treatments, the control had the highest Ni concentration in the nodules. By the R5 maturity phenological stage, nano-Ni and micro-Ni seed + foliar applications increased nodule Ni concentration by 65% and 45%, respectively, relative to the control. However, at the R7 stage, nodules in all treatments showed a decreased Ni concentration compared to the control. In contrast, Ni concentration in other tissues (stems, leaves, and roots) at all phenological stages remained higher in Ni-treated plants than in the control, particularly in plants receiving seed + foliar application. At maturity, the nano-Ni seed + foliar treatment resulted in a Ni concentration in the roots of 22 mg kg^–1^, compared to 14 mg kg^–1^ in the control. In contrast, the nano-Ni seed-only treatment had a lower Ni concentration than the control, with values of 9.6 mg kg^–1^ in the nano-Ni seed-only treatment compared to 14.1 mg kg^–1^ in the control. The macro-Ni treatment overall presented greater results compared with the control. At the R7 stage, the macro-Ni seed + foliar treatment increased Ni concentration in leaves by 45% compared to the control, while the stem showed a 2.42-fold higher Ni concentration than the control.

**Table 1:** Effect on Ni Concentrations in Soybean Tissues at Different Phenological Stages of Plants Treated with Ni-Sources, Macro, Micro, and Nanoparticles, Applied *via* Seed and Foliar Application^a^

Treatments		Ni concentration in soybean tissues (mg kg^–1^)				
Ni-sources	Ni-applications	Root	Nodule	Leaf	Stem	Grain
Flowering – R2						
Macro	Seed	7.8 ± 0.3 c	25.2 ± 2.9 a	4.2 ± 0.3 bc	2.0 ± 0.3 ab	-
Macro	Seed + Foliar	15.9 ± 0.1 ab	32.0 ± 2.5 b	5.6 ± 0.1 a	1.7 ± 0.1 b	-
Micro	Seed	20.6 ± 0.2 a	19.1 ± 1.1 c	4.6 ± 0.2 b	2.5 ± 0.2 a	-
Micro	Seed + Foliar	18.7 ± 0.3 ab	21.7 ± 0.3 bc	2.5 ± 0.3 e	1.5 ± 0.3 b	-
Nano	Seed	14.8 ± 0.2 b	18.6 ± 0.5 c	3.4 ± 0.2 d	1.9 ± 0.2 b	-
Nano	Seed + Foliar	17.3 ± 0.1 ab	22.7 ± 0.1 bc	3.7 ± 0.1 cd	1.8 ± 0.1 b	-
Control (Without Ni)		15.8 ± 0.1 b	20.1 ± 0.5 c	2.4 ± 0.1 e	0.8 ± 0.1 c	-
C.V. (%)		11.3	11.8	8.3	14.2	-
Beginning seeds – R5						
Macro		11.2 ± 0.1 d	18.9 ± 1.2 ab	2.2 ± 0.1 cd	1.1 ± 0.1 cd	-
Macro		14.3 ± 0.1 bc	14.9 ± 0.4 cd	3.3 ± 0.1 a	1.6 ± 0.1 b	-
Micro		10.2 ± 0.1 de	15.4 ± 0.4 bcd	2.9 ± 0.1 ab	0.9± 0.1 d	-
Micro		15.5 ± 0.1 ab	19.3 ± 0.4 ab	2.7 ± 0.1 abc	1.1 ± 0.1 cd	-
Nano		8.9 ± 0.2 e	18.3 ± 0.1 abc	1.3 ± 0.2 e	1.9 ± 0.2 a	-
Nano	Seed + Foliar	17.2 ± 0.3 a	22.0 ± 0.2 a	2.6 ± 0.3 bc	1.5 ± 0.3 b	-
Control (Without Ni)		13.5 ± 0.4 c	13.3 ± 3.1 d	1.7 ± 0.4 de	1.3 ± 0.4 bc	-
C.V. (%)		6.4	5.6	11.4	8.9	-
Maturity – R7						
Macro	Seed	19.7 ± 0.1 ab	21.3 ± 2.7 aAd	1.3 ± 0.1 d	0.8 ± 0.1 cd	5.2 ± 0.41 a
Macro	Seed + Foliar	19.0 ± 0.1 ab	16.8 ± 0.4 acd	1.7 ± 0.1 c	1.5 ± 0.1 a	4.5 ± 0.2 ab
Micro	Seed	17.4 ± 0.2 aAd	23.7 ± 2.3 aAd	2.2 ± 0.2 b	1.1 ± 0.2 b	4.8 ± 0.4 a
Micro	Seed + Foliar	18.7 ± 0.1 ab	30.2 ± 0.8 aCd	2.3 ± 0.1 b	1.0 ± 0.1 bc	5.3 ± 0.7 a
Nano	Seed	9.6 ± 0.1 b	25.6 ± 3.7 aAd	1.3 ± 0.1 d	0.8 ± 0.1 cd	4.1 ± 0.5 ab
Nano	Seed + Foliar	22.0 ± 0.1 a	32.6 ± 1.8 aCD	2.5 ± 0.1 a	0.9 ± 0.2 bc	4.3 ± 0.8 ab
Control (Without Ni)		14.2 ± 0.1 ab	33.6 ± 3.9 a	1.1 ± 0.1 d	0.6 ± 0.1 d	3.1 ± 0.3 b
C.V. (%)		7.0	14.6	6.9	13.1	11.2

a Values are means and standard deviations of three (R2 and R5) and five (R7) replicates. Different letters indicate significant differences according to the least difference (Tukey) test, P < 0.05.

### 3.3 Nickel Concentrations in Seeds

At R7, Ni concentrations in seeds increased across all treatments compared with the control (Table 1). The highest Ni concentration was observed with micro-Ni seed + foliar and macro-Ni seed-only applications, showing 1.68-fold and 1.73-fold increases, respectively, relative to the control. Nevertheless, the treatments did not differ significantly, except for a statistical difference compared to the control. Among the treatments, nano-Ni seed-only applications resulted in the lowest seed Ni concentration (average of 4.1 mg kg^–1^), while micro- and macro-Ni treatments averaged 4.8 mg kg^–1^ and 5.2 mg kg^–1^, respectively. In contrast, control plants had a Ni concentration of 3.08 mg kg^–1^ in seeds (Table 1).

### 3.4 Assessment of Urease, Nitrate Reductase Activities in Leaves and Nitrogenase Activity in Nodules

Urease activity (Figure 4a, b) and nitrate reductase (NR) activity (Figure 4c, d) were evaluated in vivo at two time points: 15 days after combined seed and foliar Ni treatment (V6 stage) and at the R2 phenological stage. Urease activity (Figure 4a,b) increased significantly with Ni application, regardless of whether it was applied through seed or seed + foliar. Plants in the control group that did not receive Ni presented the lowest urease activity. Seed + foliar application of Ni enhanced urease activity during both periods compared to the seed-only Ni application, with the peak activity occurring at the R2 stage, which is in full bloom (Figure 4a). In the first evaluation period, which occurred 15 days after foliar Ni application, plants treated with macro- and micro-Ni showed higher urease activity than those treated with nano-Ni in the seed + foliar treatment. In contrast, for the seed-only treatment, nano-Ni exhibited greater urease activity compared to both micro- and macro-Ni treatments. At the R2 phenological stage, there was no significant increase in urease activity among the treatments, except when compared to the control plants. The highest urease activity recorded was at R2 in the treatment combining nano-Ni seed + foliar application, measuring 12.2 μmol of N-NH_4_^+^ gfw^–1^ h^–1^ (Figure 4b). In contrast, the control treatment yielded the lowest urease activity, averaging 10.7 μmol of N-NH_4_^+^ gfw^–1^ h^–1^ at R2.

**Figure 4:**
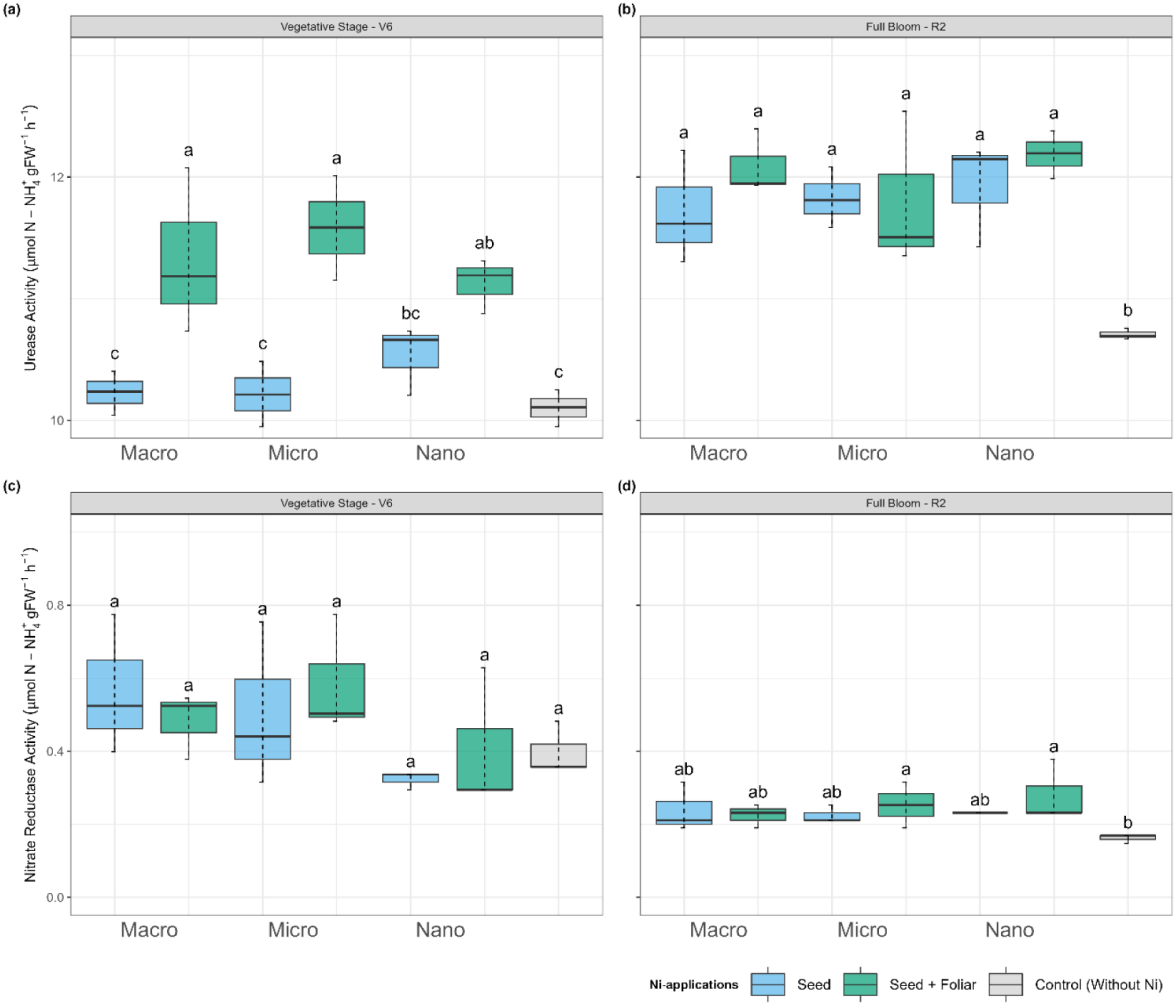
Effect on (a,b) urease and (c,d) nitrate reductase activity in soybean leaves at (a,c) vegetative stage (V6) and (b,d) flowering (R2) of plants treated with Ni sources—macro, micro, and nanoparticles—applied via seed and foliar. Values are means and standard deviations of three replicates. Different letters indicate significant differences according to the least difference (Tukey) test, *P* < 0.05. Combined letters (e.g., “bc”) indicate overlap between statistical groups.

At the V6 growth stage, plants treated with nano-Ni exhibited the lowest nitrate reductase (NR) activity. The nano-Ni treatment reduced NR activity by 41% and 43% relative to the macro- and micro-Ni treatments, respectively, during this period, regardless of the Ni application (seed-only or seed + foliar). At the R2 growth stage, there were no significant differences in NR activity among the treatments, except for the control group. Plants treated with micro-Ni seed + foliar exhibited the highest NR activity, followed by macro-Ni seed-only. At the R2 phenological stage, plants treated with nano-Ni, especially those receiving seed + foliar, exhibited the highest NR activity. The nano-Ni seed + foliar treatment was 1.42-fold greater than the control plant, without any Ni application at the R2 phenological stage. On average, plants that received Ni through seed-only treatment or a combination of seed + foliar applications exhibited a 1.49-fold increase in NR activity compared with control plants.

The maximum nitrogenase activity, measured as the acetylene reduction activity (ARA), was observed in plants treated with macro-Ni applied via seed at the R2 phenological stage. This treatment enhanced nitrogenase activity by 1.85-fold relative to the control, followed by the micro-Ni treatment with seed + foliar application, which showed a 1.75-fold increase compared to the control (Figure 5a). Conversely, nano-Ni application via seed reduced nitrogenase activity by approximately 33% relative to the control and 20% relative to nano-Ni applied via seed + foliar. At the R5 phenological stage (Figure 5b), ARA activity decreased by approximately 70% compared with the R2 stage, indicating a significant decline in nitrogenase performance as the plants advanced through their growth stages. At R5, the control plants had greater ARA activity compared to the treatments. The lowest ARA activity in R5 was recorded in the treatment micro-Ni seed + foliar, with a value of 13.4 μmol h^–1^ and the control plants had a value of 70 μmol h^–1^.

**Figure 5:**
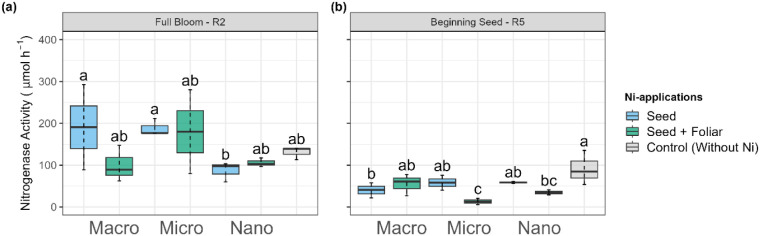
Effect on nitrogenase activity in the root nodules of soybean at (a) flowering (R2) and (b) beginning seeds (R5) of plants treated with Ni sources—macro, micro, and nanoparticles—applied via seed and foliar. Values are means and standard deviations of three replicates. Different letters indicate significant differences according to the least difference (Tukey) test, *P* < 0.05. Combined letters (e.g., “bc”) indicate overlap between statistical groups.

### 3.5 Nodule Dry Weight and Number of Nodules

Nodulation parameters, including the quantity and dry biomass of nodules, exhibited significant variation across treatments and phenological stages, with the most pronounced results observed at maturity (Figure 6). At the stage of R2 (Figure 6a), nano-Ni application via seed increased the dry weight of nodules by 2.4- and 2.2-fold relative to the macro- and micro-Ni treatments, respectively, and 1.35-fold compared to the control plants. Similarly, the number of nodules was highest in plants receiving nano-Ni via seed application at R2. At R2, the macro-Ni treatment with seed + foliar application resulted in the highest number and dry weight of nodules per plant, followed by nano-Ni. At the R5 growth stage, control plants exhibited the highest average nodule count, with 480 nodules per pot. This was followed by plants treated with nano-Ni applied via seed + foliar methods, which had an average of 380 nodules per pot. However, the highest nodule dry weight was observed in the macro-Ni seed-only treatment, with a value of 3.64 g. The nano-Ni treatment had a nodule dry weight of 3.5 g, while control plants averaged 2.01 g at R5. As the plants matured, those treatments with nano-Ni exhibited a decline in the nodulation parameters across the phenological stages (Figure 6c; f). At the R7 maturity stage, macro-Ni seed-only treatments outperformed other treatments, yielding nodule dry weights 1.81 and 1.75 times higher than those of nano-Ni and control plant treatments, respectively. The findings emphasize the significant influence of both the Ni source and its application method on nodulation. Macro-Ni treatments demonstrated superior performance at the R7 stage, while nano-Ni applications, whether via seed or a combination of seed + foliar methods, were particularly effective during earlier stages (R2 and R5). This suggests that nano-Ni has the potential to enhance nodulation and BNF dynamics in the initial growth phases.

**Figure 6:**
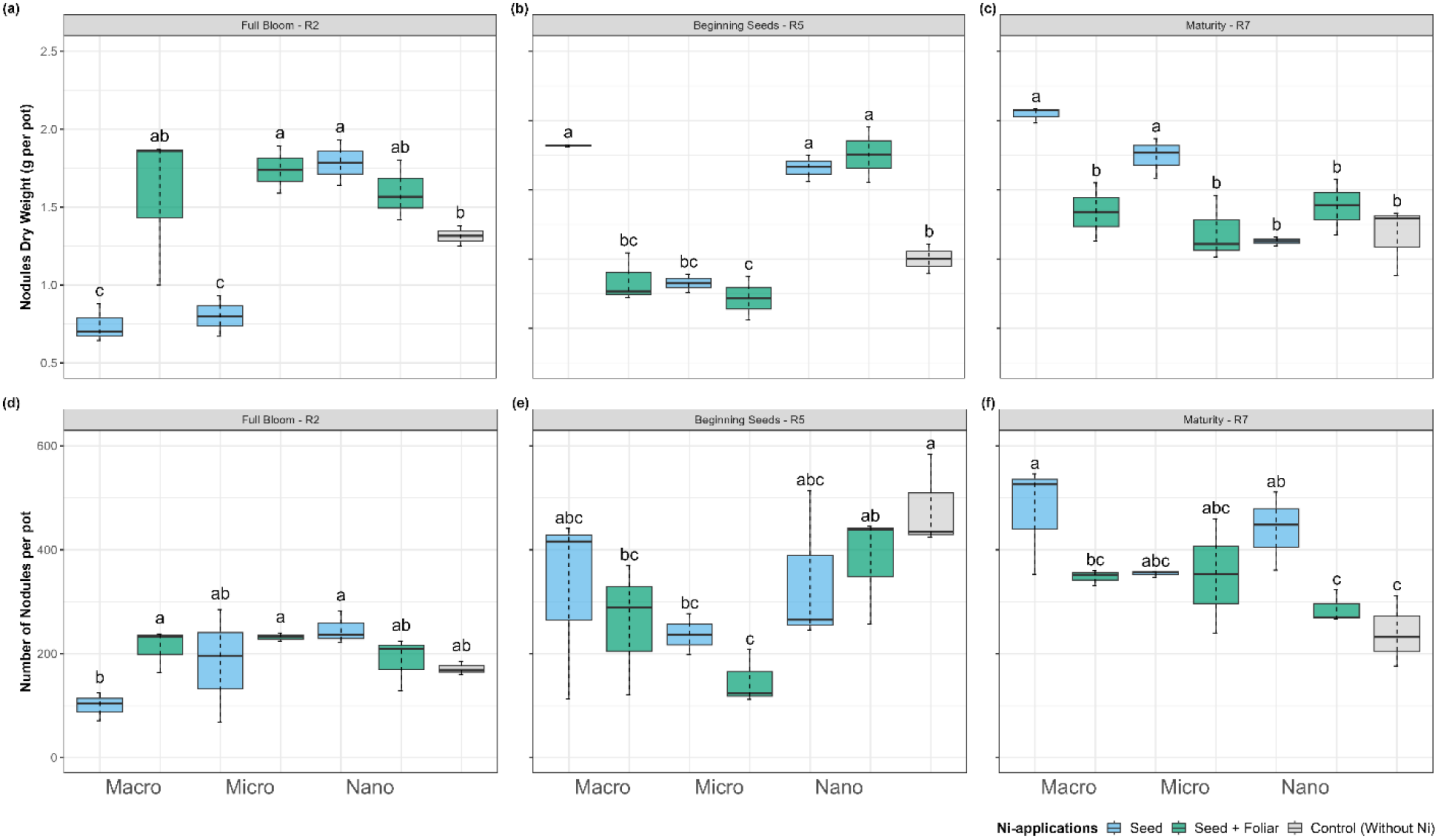
Effect on dry weight (a-*b*-c) and number of nodules (d-e-f) in soybean at (a,d) flowering (R2), (b,e) beginning seeds (R5), and (c,f) maturity (R7) of plants treated with Ni sources—macro, micro, and nanoparticles—applied via seed and foliar. Values are means and standard deviations of three (R2 and R5) and five (R7) replicates. Different letters indicate significant differences according to the least difference (Tukey) test, *P* < 0.05. Combined letters (e.g., “bc”) indicate overlap between statistical groups.

### 3.6 Observations on Plant Growth and Visibly Greener Leaves

Figure 7 illustrates the visible differences in plant growth among treatments 4 days after foliar Ni application at the V4 stage. Compared to seed-only Ni treatments, the combination of seed + foliar Ni application resulted in visibly greener leaves and greater biomass accumulation. Notably, plants receiving nano-Ni via both seed and foliar application exhibited more pronounced greening and increased leaf biomass compared to those that received Ni exclusively through seed treatment.

**Figure 7:**
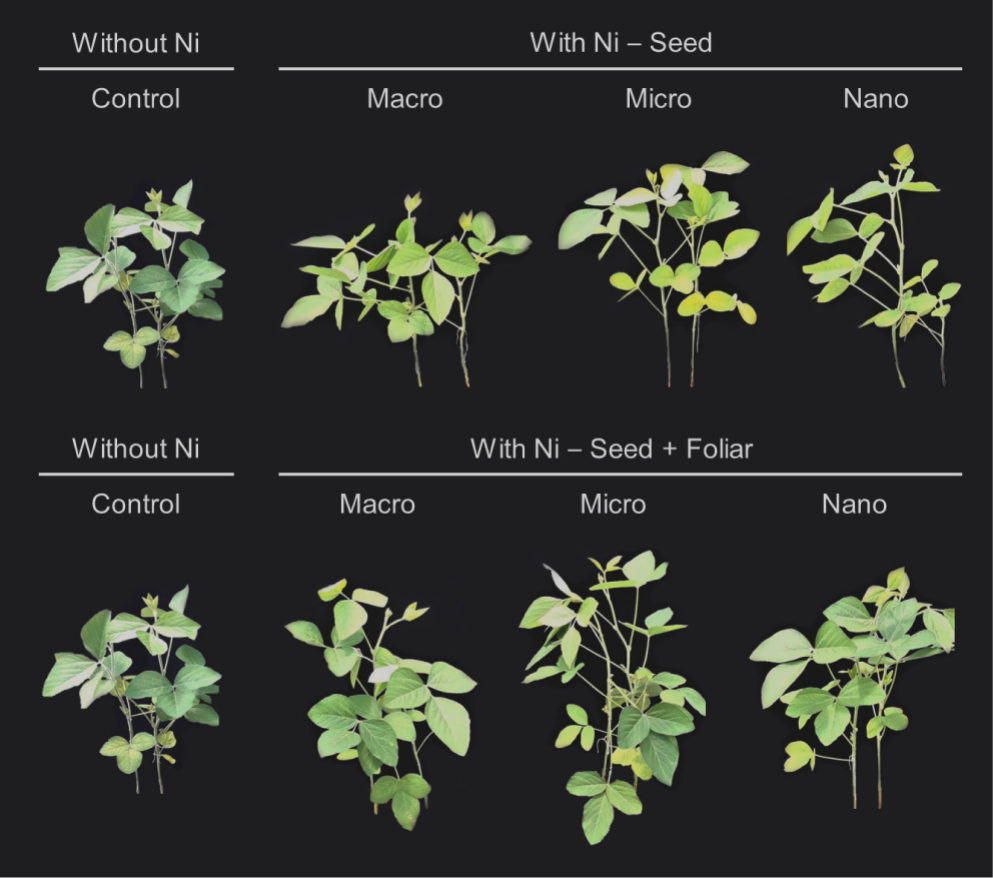
Effect on soybean growth at the vegetative stage (V6) of plants treated with Ni sources—macro, micro, and nanoparticles—applied via seed and foliar. Plants were grown in pots containing 3 kg of soil (≈18 cm diameter).

### 3.7 Ammonia, Nitrate, and Total Ureides Concentration in Soybean Tissues

The ammonia concentration, indicative of urease activity, was positively influenced by the Ni supply in most treatments, particularly at the R2 stage (Figure 8). The macro- and micro-Ni that received Ni only via seed had the lowest ammonia results compared to the control plants, averaging 223, 233, and 348 μmol g^–1^ DW, respectively. At the R2 growth stage, the seed + foliar Ni application increased ammonia levels significantly in the stem tissues across macro- and micro-Ni treatments, apart from the nano-Ni seed + foliar treatment, which exhibited a 43% reduction compared to the Ni-seed treatment, measuring 275.1 and 481.4 μmol g^–1^ DW, respectively (Figure 8a). Conversely, at the R5 stage, the application of seed + foliar nano-Ni resulted in the highest concentration of ammonia, exceeding macro- and micro-Ni treatments by 1.7-fold and 1.22-fold, respectively. By the time the plants reached maturity, the ammonia concentration from the seed + foliar nano-Ni application was lower than that from the nano-Ni application via seed only, measuring 333 μmol g^–1^ DW compared to 573 μmol g^–1^ DW. Notably, the concentration from the seed-only application was the highest among all treatments (Figure 8c).

**Figure 8:**
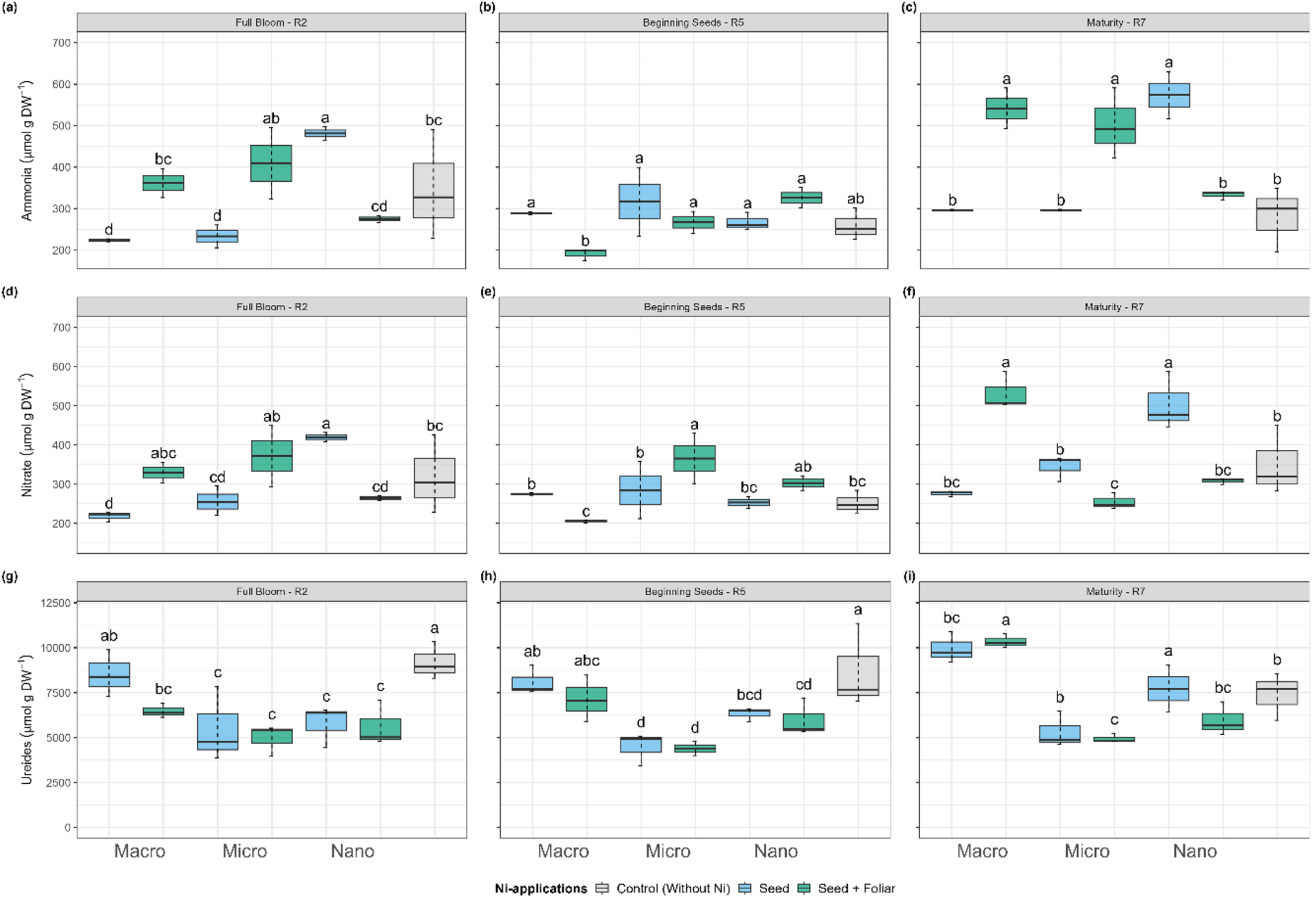
Effect on (a-b-c) ammonia, (d-e-f) nitrate, and (g-h-i) ureides concentration in soybean stems at (a-d-g) flowering (R2), (b-e-h) beginning seeds (R5), and (c-f-i) maturity (R7) of plants treated with Ni sources—macro, micro, and nanoparticles—applied via seed and foliar. Values are means and standard deviations of three (R2 and R5) and five (R7) replicates. Different letters indicate significant differences according to the least difference (Tukey) test, *P* < 0.05. Combined letters (e.g., “bc”) indicate overlap between statistical groups.

A similar trend was observed in the nitrate concentrations of soybean tissues. The application of Ni through both seed and foliar methods generally increased nitrate levels, except for the nano treatment. At the R2 stage, plants receiving only the seed Ni applications showed nitrate concentrations of 218 μmol g^–1^ DW for macro-Ni, 256 μmol g^–1^ DW for micro-Ni, and 420 μmol g^–1^ DW for nano-Ni. In contrast, the seed + foliar application resulted in nitrate levels of 330 μmol g^–1^ DW for macro-Ni, 371 μmol g^–1^ DW for micro-Ni, and 264 μmol g^–1^ DW for nano-Ni (see Figure 8d). By the R5 stage, the nitrate levels for seed-only Ni treatments were 275 μmol g^–1^ DW for macro-Ni, 285 μmol g^–1^ DW for micro-Ni, and 253 μmol g^–1^ DW for nano-Ni. In comparison, the seed + foliar treatments yielded values of 205 μmol g^–1^ DW for macro-Ni, 365 μmol g^–1^ DW for micro-Ni, and 302 μmol g^–1^ DW for nano-Ni (see Figure 8e). At the R7 stage, nitrate concentrations peaked in the nano seed-only treatment at 503 μmol g^–1^ DW. Meanwhile, the macro and micro treatments applied via seed + foliar methods reached nitrate concentrations of 532 μmol g^–1^ DW and 254 μmol g^–1^ DW, respectively (Figure 8f).

The results reveal that the Ni source and application method distinctly influenced ammonia and nitrate accumulation in soybean tissues across phenological stages. Seed + foliar nano application had mixed outcomes, reducing ammonia and nitrate levels at R2 but boosting ammonia concentration at R5. Macro and micro treatments generally maintained consistent benefits across stages, highlighting their potential for improving nitrogen metabolism in soybean cultivation. The total concentration of ureides (allantoin and allantoic acid) was influenced by the sources of Ni and the methods of application (Figure 8g–i). In the control treatment (without Ni application), ureide concentrations were higher during the early phenological stages compared with the Ni-supplied treatments. However, at the maturity stage (R7), plants treated with Ni showed greater total ureide levels than the control plants, indicating an improvement in nitrogen metabolism. Unexpectedly, the combination of seed + foliar Ni application did not consistently increase ureide concentration compared to treatments where Ni was applied only via seed. At R7, the ureide levels in plants treated with seed-only Ni were as follows: 9946 μmol g^–1^ DW for macro-Ni, 5318 μmol g^–1^ DW for micro-Ni, 7724 μmol g^–1^ DW for nano-Ni, and 7411 μmol g^–1^ DW for the control. In contrast, the concentrations for the seed + foliar Ni application were 10345 μmol g^–1^ DW for macro-Ni, 4943 μmol g^–1^ DW for micro-Ni, and 5955 μmol g^–1^ DW for nano-Ni. These results suggest that while Ni application enhances total ureide accumulation in later stages, both the method of application and the source of Ni significantly influence the degree of this response. Seed-only Ni treatments displayed more consistent benefits, especially for macro- and nano sources, compared to the combination of seed and foliar applications.

### 3.8 Total Nitrogen Concentration in Soybean Tissues

The concentrations of total N in plant tissues are presented in Tables 2 and 3. At the R2 stage, the highest N content was found in plants treated with nano-Ni applied via seeds, averaging 44.8 g kg^–1^. In contrast, the lowest N content was observed in plants exposed to micro-Ni through seed + foliar application, averaging 33.2 g kg^–1^. At the R5 stage, the highest N concentration was observed in plants that received both seed + foliar applications of nano-Ni, which had an N content of 37 g kg^–1^. Conversely, the lowest N content was again noted in the micro-Ni seed + foliar-treated plants at 26.8 g kg^–1^. During the R7 stage (maturity stage), the trend remained consistent with the R5 stage: the highest N content was found in plants treated with nano-Ni via seed + foliar, exhibiting a value of 32.4 g kg^–1^, while the lowest was again in the micro-Ni treatment seed + foliar, at 27.7 g kg^–1^. Overall, except for the micro-Ni treatments, the combination of seed and foliar applications significantly boosted N content, particularly during the R5 and R7 stages.

**Table 2:** Effect on Natural Abundance of δ^15^N‰, Estimation of Biological N_2_ Fixation (BNF), and N-Total in the Soybean (Aerial Parts) at Different Phenological Stages of Plants Treated with Ni-Sources, Macroparticles, Microparticles, and Nanoparticles, Applied via Seed and Foliar Application^a^

Treatments		Aerial parts of soybean plants		
Ni-sources	Ni-applications	δ^15^N‰	BNF (%)	N-total(g kg^–1^)
Full Bloom – R2				
Macro	Seed	-0.7 ± 0.2 ab	68.7 ± 3.4 bc	39.9 ± 2.8 abc
Macro	Seed + Foliar	-1.5 ± 0.4 bc	81.4 ± 6.4 ab	41.7 ± 0.4 ab
Micro	Seed	-1.3 ± 0.1 abc	77.1 ± 7.7 abc	38.3 ± 0.5 bcd
Micro	Seed + Foliar	-1.3 ± 0.2 c	87.4 ± 3.6 a	33.2 ± 2.6 d
Nano	Seed	-0.2 ± 0.5 a	60.5 ± 8.4 c	44.8 ± 2.0 a
Nano	Seed + Foliar	-0.8 ± 0.1 abc	70.8 ± 1.3 abc	35.0 ± 0.1 cd
Control (Without Ni)		-1.2 ± 0.2 abc	77.3 ± 2.5 abc	39.2 ± 1.3 bc
C.V. (%)		20.9	6.9	7.3
Beginning seeds – R5				
Macro	Seed	-1.3 ± 0.3 a	79.4 ± 4.4 a	37.6 ± 0.10 a
Macro	Seed + Foliar	-0.7 ± 0.8 a	68.6 ± 13.5 a	38.1 ± 0.02 a
Micro	Seed	-1.5 ± 0.2 a	81.8 ± 3.9 a	34.9 ± 0.8 ab
Micro	Seed + Foliar	-1.9 ± 0.1 a	87.9 ± 1.1 a	26.8 ± 0.8 c
Nano	Seed	-1.2 ± 0.1 a	77.5 ± 6.6 a	30.8 ± 2.8 bc
Nano	Seed + Foliar	-1.5 ± 0.4 a	81.6 ± 7.0 a	37.0 ± 1.9 a
Control (Without Ni)		-1.1 ± 0.6 a	74.9 ± 10.1 a	34.2 ± 2.2 ab
C.V. (%)		16.3	6.4	7.9
Maturity – R7				
Macro	Seed	-2.0 ± 0.3 ab	90.1 ± 5.1 a	29.4 ± 0.2 b
Macro	Seed + Foliar	-1.3 ± 0.6 ab	88.7 ± 0.32 ab	33.2 ± 1.7 a
Micro	Seed	-1.7 ± 0.4 ab	85.5 ± 7.0 ab	28.7 ± 0.4 b
Micro	Seed + Foliar	-2.1 ± 0.1 b	92.3 ± 0.3 a	27.7 ± 1.0 b
Nano	Seed	-2.0 ± 0.2 ab	90.1 ± 4.5 a	27.8 ± 0.4 b
Nano	Seed + Foliar	-0.6 ± 0.8 a	67.3 ± 13.3 b	32.4 ± 0.4 a
Control (Without Ni)		-0.8 ± 0.5 ab	70.4 ± 9.0 ab	28.8 ± 0.7 b
C.V. (%)		9.4	5.5	5.3

a Values are means and standard deviations of three (R2 and R5) and four (R7) replicates. Different letters indicate significant differences according to the least difference (Tukey) test, P < 0.05.

**Table 3:** Effect on Natural Abundance of δ^15^N‰, Estimation of Biological N_2_ Fixation (BNF), and N-Total in the S of Soybean at Phenological Stage of Maturity (R7) of Plants Treated with Ni-Sources, Macro, Micro, and Nanoparticles, Applied *via* Seed and Foliar Application^a^

Treatments		Grains of soybean plants		
Ni-sources	Ni-applications	δ ^15^N‰	BNF (%)	N-total(g kg^–1^)
Maturity – R7				
Macro	Seed	-0.2 ± 0.1ab	61.1 ± 2.0 ab	48.6 ± 0.3 c
Macro	Seed + Foliar	-0.3 ± 0.2 ab	61.7 ± 3.7 a	56.8 ± 0.5 a
Micro	Seed	-0.1 ± 0.0 ab	63.1 ± 4.5 a	52.7 ± 0.3 abc
Micro	Seed + Foliar	-0.12 ± 0.3 ab	58.1 ± 1.4 ab	52.6 ± 0.3 abc
Nano	Seed	-0.4 ± 0.2 b	58.7 ± 1.2 ab	55.1 ± 0.6 ab
Nano	Seed + Foliar	-0.1 ± 0.0 ab	59.5 ± 4.9 ab	54.0 ± 1.2 ab
Control (Without Ni)		0.16 ± 0.1 a	53.2 ± 1.6 b	51.8 ± 3.9 bc
C.V. (%)		56.8	4.7	2.4

a Values are means and standard deviations of three (R2 and R5) and five (R7) replicates. Different letters indicate significant differences according to the least difference (Tukey) test, P < 0.05.

### 3.9 BNF Evaluation Using δ^15^N‰ Technique

The natural δ^15^N abundance method was used to estimate BNF in the aerial parts (Table 2) and seeds (Table 3) of soybean crops across multiple key phenological stages: R2, R5, and R7. The nonfixing plant species *Oryza sativa* (control) showed a δ^15^N‰ of 3.5‰, providing a baseline for comparison. At the R2 stage, differences were found among the treatments. Soybean plants treated with macro-Ni (via seed + foliar) had a δ^15^N‰ value of −1.5, while those treated with micro-Ni had −1.3, and plants with nano-Ni applied via foliar showed a δ^15^N‰ value of −0.8. Notably, the BNF values ranged from 70.8% (nano-Ni) to 87.4% (micro-Ni), with macro-Ni seed + foliar application achieving 81.4% BNF at R2. The control plants, with no Ni application, showed a BNF of approximately 77.3% at R2. At the R5 stage, there were no significant differences among treatments, but the macro-Ni via seed + foliar plants exhibited a lower BNF compared to the treatments with Ni. Similar results were observed at the R7 stage, with the control treatment showing a BNF of around 70.4%, while all treatments with Ni had around 98% BNF, except for nano-Ni seed + foliar that exhibited a BNF value of 67.3%.

### 3.10 Nitrogen in Seeds Derived from the Atmosphere (BNF)

The analysis of δ^15^N‰ in the seeds indicated that approximately 60% of the nitrogen present in the seeds was derived from biological nitrogen fixation, compared with 53% in the untreated control (Table 3). In general, Nil supplementation, independent of source or application method (seed alone or seed combined with foliar treatment), promoted BNF and elevated nitrogen accumulation in both aboveground tissues and seeds. The combined seed and foliar Ni treatment resulted in the highest BNF contribution and total nitrogen levels, emphasizing the beneficial role of Ni in regulating nitrogen metabolism in soybean.

### 3.11 Correlations and Principal Component Analysis (PCA) of Ni Treatments in Soybean

#### 3.11.1 Phenological Stage R2 (At Flowering Stage)

The Pearson’s correlation coefficients revealed key relationships (Suppl. Figure 1a), with positive correlations: (i) urease activity with leaf and stem dry weight; (ii) nitrate reductase activity with nitrogenase activity; (iii) nodules (number and dry weight) with stem, leaf, and root dry weight; (iv) Ni concentration in roots with the number of nodules and BNF; and (v) BNF with nitrogenase activity; (vi) ammonia and nitrate concentration with nodules’ dry weight. On the other hand, the following negative correlations were observed: (i) ureide levels associated with nodule abundance, nitrate reductase activity, and Ni concentration in the roots; (ii) nitrogenase activity with leaf dry weight. The PCA (Suppl. Figure 2a) was performed to evaluate the relationships among treatments. The first two principal components (PC1 and PC2) explained 26.88% and 15.64% of the total variance, respectively. PC1 primarily separated treatments based on biomass accumulation and Ni concentration. Positive loadings on PC1 were associated with higher nodulation parameters (Number Nod, Nod DW), greater BNF, increased plant biomass (Leaf DW, Stem DW, Roots DW), and higher Ni accumulation in tissues ([Ni] Leaf, [Ni] Nod, [Ni] Stem, [Ni] Roots). Conversely, negative loadings on PC1 were linked to higher ureide concentrations, nitrogenase activity, and δ^15^N, suggesting lower nitrogen fixation efficiency and a greater reliance on soil nitrogen. PC2 separated treatments based on enzyme activity. Variables positively correlated with PC2 included nitrate reductase (N. reductase) and urease activity, indicating an increased enzymatic role in nitrogen metabolism. In contrast, negative loadings on PC2 were associated with nitrogenase activity and ureide concentrations. The clustering pattern observed in the PCA biplot was further confirmed by hierarchical clustering (Suppl. Figure 2b), where nanofertilizer treatments grouped separately from macro- and micro-treatments, suggesting distinct nitrogen assimilation pathways. Control treatments (without nickel) clustered together, indicating lower nitrogen fixation efficiency and reduced biomass accumulation.

#### 3.11.2 Phenological Stage R5 (At Seeds Set)

The Pearson’s correlation coefficients (Suppl. Figure 1b) revealed key relationships, with positive correlations as follows: (i) nitrate and ammonia; (ii) leaf DW and total N; (iii) number of nodules and nodules dry weight; and (iv) anodule number and leaf dry weight. In contrast negative correlations were as follows: (i) Ni concentration in the nodules and nitrate and (ii) nitrogenase activity with root Ni concentration. The PCA (Suppl. Figure 2c) was performed to evaluate the relationships among treatments. The first two principal components (PC1 and PC2) explained 24.55% and 19.43% of the total variance, respectively. PC1 was primarily associated with BNF, nitrate, ammonia, and root dry weight, with positive loadings for these variables. In contrast, δ^15^N and urease exhibited strong negative loadings. PC2 was positively associated with Ni concentration in leaves and roots, while negatively correlated with biomass-related traits such as stem and leaf dry weight. The PCA biplot revealed clear grouping patterns among treatments. Nano treatments, regardless of the application, were positioned along the positive PC1 axis. Macro treatments and control plants clustered toward the negative PC1 axis. The clustering pattern observed in the PCA biplot was further confirmed by hierarchical clustering (Suppl. Figure 2d), which showed distinct treatment groupings. Nano treatments formed a separate cluster, while micro treatments grouped; and macro treatments grouped together, distinct from macro and control treatments.

#### 3.11.3 Phenological Stage R7 (At Maturity)

The Pearson’s correlation coefficients revealed key relationships (Suppl. Figure 1c), with positive correlations as follows: (i) BNF in the grains with nodules dry weight, Ni concentration in the stem, and number of grains; (ii) BNF and Ni concentration in the grains; (iii) ammonia concentration with total N in the grain and number of grains; (iv) Ni concentration in roots with nodules dry weight, BNF, and number of nodules; and (v) Ni concentration in the roots with leaf dry weight. The negative correlations were as follows: (i) δ^15^N‰ with number of nodules and ammonia concentration; (ii) Ni concentration in the roots with N total in the grain; (iii) ammonia concentration with nodules dry weight; and (iv) ureides with Ni concentration in the leaf. The PCA (Suppl. Figure 2e) was performed to evaluate the relationships among treatments. The first two principal components (PC1 and PC2) accounted for 20.47% and 15.98% of the total variance, respectively. PC1 was primarily influenced by δ^15^N grains, Ni concentration in the nodules, and BNF, while PC2 was largely associated with nitrate, ammonia, and Ni concentration in the leaf. Treatments were distributed along both axes, with nano treatments (seed-only, seed + foliar) separating distinctly from the control and macro treatments. The PCA biplot shows that BNF grains, the number of nodules, and the number of ureides are closely clustered together. Conversely, δ^15^N and Ni concentration in the nodules were negatively associated with BNF. The hierarchical clustering (Suppl. Figure 2f) further delineated treatment effects, forming three major groups. Micro seed-only and seed + foliar clusters were clustered together, nano seed-only and control were clustered together, while macro seed-only and seed + foliar formed a distinct cluster.

## 4 Discussion

This study shows that Ni applications, *via* seed treatment and foliar spray, distinctly modulated biochemical, nutritional, and physiological processes in soybeans, upregulating N-metabolism and BNF. The findings emphasize the different impacts of Ni sources (nano, micro, and macro-Ni forms) and application methods, presenting a potential mechanistic narrative for the role of Ni in legume nutrient optimization. The performance of the nano-Ni and micro-Ni treatments, especially with seed + foliar applications, raises important questions about how particle size influences Ni bioavailability and its integration into plant metabolism. Although increases in urease and NR activity were observed, the results indicate that Ni application, regardless of particle size or mode of application, enhances the activity of these enzymes’ cofactors.^60^

This study explores the combined effect of seed-applied Ni and supplementary foliar Ni application, focusing particularly on the use of nanoparticles. While previous research has examined the effects of Ni oxide nanoparticles (NiO-NPs) on barley, wheat, and tomato,^93,94^ this is the first investigation into the impacts of Ni hydroxide nanoparticles (Ni­(OH)_2_) and the form of Ni application on soybean plants. This study demonstrated that nano-Ni seed + foliar supplementary application of Ni resulted in greater Ni accumulation in roots and nodules relative to seed treatments alone, mainly at an early stage. Notably, plants treated with nano-Ni via seed exhibited a decline in nodule Ni concentration as the soybean plants developed, unlike those treated with macro-Ni, which maintained higher concentrations. However, the combined seed + foliar nano-Ni application ensured consistent or increased Ni concentrations in plant tissues throughout the developmental stages, highlighting its efficacy in promoting Ni translocation and accumulation in critical organs. This aligns with previous studies indicating that Ni-NPs tend to accumulate more in roots and nodules than in shoots, as observed in barley, wheat, and other plants.^94–97^ Additionally, our findings indicate that despite the expected slow release of Ni nanoparticles across developmental stages, the measured parameter increased during early stages and declined at later stages. The precise mechanisms driving this temporal pattern remain unclear; possible explanations include transiently enhanced bioavailability or early physiological stimulation, followed by nanoparticle transformation, sequestration, or toxicity at later stages.

In line with these possibilities, this response may also be attributed to the physicochemical properties of the α-Ni­(OH)_2_ nanoparticles, including their nanometric size, high surface area, and hydrated α-phase structure, which favor enhanced Ni availability during early growth stages. Previous studies report particle sizes in the 1–10 nm range and increased reactivity of the α-Ni­(OH)_2_ phase, promoting partial dissolution under acidic soil conditions.^66,67,98^

Under these conditions, soil pH emerges as a key factor controlling Ni availability from α-Ni­(OH)_2_ nanoparticles. Given the low solubility product of nickel hydroxide (Ksp = 5.5 × 10^–16^), these nanoparticles remain largely insoluble under neutral to mildly alkaline conditions but become readily soluble under acidic conditions (pH < ∼4), generating hydrated Ni^2+^ ions.^66^

In the context of the acidic nature of Brazilian soils and the historically low pH of the soil used in this study (∼4.2), such dissolution behavior is particularly relevant for interpreting temporal changes in Ni availability and plant responses. Previous studies have demonstrated that soil acidity strongly influences metal oxide nanoparticle behavior; for example, ZnO nanoparticles have been reported to exhibit up to a 200-fold increase in Zn solubility under acidic compared with alkaline conditions, resulting in dose-dependent phytotoxicity.^99^ Additionally, soil pH has been shown to significantly affect the distribution of Zn derived from ZnO nanoparticles among different soil particle-size fractions.^100^ Although soil pH was adjusted with lime prior to the experiment, BNF by soybean can induce localized rhizosphere acidification through proton release.^101^ Such pH changes may influence nanoparticle dissolution and metal availability over time. While soil or rhizosphere pH was not measured at the end of the experiment, this process represents a plausible mechanism contributing to the observed temporal shift in plant responses. In this context, as soybean development progressed, the initial positive effects diminished, likely reflecting a mismatch between sustained Ni release and declining plant Ni demand. Given that Ni is required in only trace amounts, continued availability beyond early growth stages may lead to nutrient imbalance or interference with other micronutrients. Additionally, strong interactions between α-Ni­(OH)_2_ nanoparticles and soil mineral components may create localized zones of elevated Ni availability, further contributing to the observed decline in growth responses at later stages due to toxicity. Such effects are consistent with the preferential association of predominantly negatively charged nanoparticles with clay minerals, particularly under acidic conditions, where electrostatic interactions are enhanced.^67,102,103^ The aboveground parts of the plants (stem and leaves) showed that the application of nano-Ni and macro-Ni, both as seed + foliar treatments, led to greater Ni accumulation compared with seed-only treatment. However, in the case of the micro-Ni treatment, the application of Ni solely to the seeds yielded better results (Table 1). In addition, the combined seed and foliar application of Ni significantly enhanced its accumulation in soybean seeds. Nano and micro-Ni fertilizers, applied via seed and foliar spray, were particularly effective, corroborating findings from previous studies (e.g., Kutman et al., 2013, 2014).^31^ Higher Ni concentrations in nodules emphasize its essential role in BNF, particularly for the hydrogenase enzyme crucial for hydrogen recycling.^104^ The observed differences in nodule Ni concentrations across the treatments offer additional mechanistic insights. Nano-Ni-treated plants showed lower initial nodule Ni concentration relative to macro- and micro-Ni forms but maintained or increased concentration during later stages (Table 1), indicating a more consistent nutrient supply. This pattern indicates that nano-Ni promotes a gradual accumulation of Ni in nodules, enhancing their functionality over time; nanoparticles can supply nutrients to the plant more gradually and with greater control than sulfate fertilizers.^43,52^ However, the lower nodule Ni concentrations in some nano-Ni treatments at early stages raise questions about potential trade-offs between rapid nutrient availability and sustained delivery. Addressing these dynamics could further enhance the design of nanofertilizers for legume crops.

Interestingly, the Ni concentration in leaves varied throughout the phenological stages (Table 1). At the R2 stage, plants treated with nano-Ni, whether via seed or seed + foliar application, showed lower Ni concentrations in leaves relative to other sources, especially macro-Ni. For example, leaves from plants treated with macro-Ni via seed + foliar application had an average Ni concentration of 5.6 mg kg^–1^, while those treated with nano-Ni via seed + foliar application averaged 3.7 mg kg^–1^. However, by the R7 stage, soybean leaves treated with nano-Ni exhibited increased Ni concentrations, surpassing those of plants treated with macro-Ni via seed + foliar application. The observed increase in Ni concentration in leaves over time following nano-Ni application is likely due to the gradual and controlled nutrient release typical of nanofertilizers, combined with the low water solubility of Ni­(OH)_2_.^53,105^ This controlled release likely explains the higher Ni concentrations in leaves at later developmental stages, ensuring efficient nutrient utilization throughout plant growth. The reduced seed Ni concentration with nano-Ni treatments compared to those with macro- and micro-Ni forms may indicate a trade-off between immediate physiological benefits and long-term nutrient storage. This phenomenon warrants further exploration to optimize Ni formulations for both agronomic performance and food safety.^106^

This study showed that seed + foliar Ni application, especially with nanoparticles, stimulated BNF, with the greatest impact observed during the R5 and R7 phenological stages (Tables 2 and 3) when atmospheric N assimilation peaked, as indicated by δ^15^N values approaching zero. Similarly,^18^ it was reported that BNF increased by 12% using seed-applied Ni. The evaluation of BNF using δ^15^N analysis provides compelling evidence of the critical role of Ni in nodule function and atmospheric N assimilation. Combined seed + foliar applications significantly boosted BNF efficiency, with smaller Ni particles (nano and micro-Ni) treatments achieving the highest contributions during reproductive stages. This enhancement is likely linked to Ni function in activating hydrogenase, an enzyme that improves nitrogenase efficiency by eliminating hydrogen byproducts. These findings suggest that nano-Ni fosters optimal nodule functionality and hydrogenase activity, likely through its interaction with nitrogenase during critical growth phases, potentially mitigating micronutrient deficiencies commonly found in tropical soils. Nitrogenase activity peaked at R2, consistent with maximum nodulation during this stage.^69^ While seed-applied micro-Ni initially boosted nitrogenase activity, foliar applications were more effective later, reflecting developmental shifts in nutrient demand and differing solubility rates of Ni sources. These findings align with reports of nanofertilizers delivering nutrients more efficiently than conventional salts.^57,107^ This enzymatic enhancement is likely linked to the role of Ni in activating urease and hydrogenase enzymes, which are critical for the BNF process. The delayed peak in NR activity with nano-Ni treatments compared to those with macro- and micro-Ni forms suggests a unique temporal modulation of N metabolism. This mechanistic distinction highlights the potential for nano-Ni formulations to sustain enzymatic activity over extended periods, a feature critical for crops in nutrient-low availability soils.^21,108,109^

Our results show a clear stage-dependent response of soybean N cycling to Ni fertilization, especially when Ni is supplied in nanoparticle form. At the beginning seed stage (R5), the correlation matrix revealed strong positive relationships among the classical indicators of biological N acquisition, such as biological nitrogen fixation (BNF), ureides, total N, and nodulation traits, while δ^15^N was negatively correlated with BNF, consistent with its well-known role as an inverse proxy for symbiotic N_2_ fixation. Notably, the concentrations of Ni in stem, leaf, and root tissues were only weakly or inconsistently correlated with these N-related traits, indicating that the simple accumulation of Ni does not directly translate into enhanced N fixation. Instead, the effects of Ni are likely mediated through specific metabolic pathways, such as urease and nitrate-reductase activity, rather than through total tissue Ni concentration alone.

The principal component analysis (PCA) further highlights a temporal shift in the plant response. At the early reproductive stage (R2), the Ni-nanoparticle treatments clustered with vectors representing high N metabolic activity (urease, nitrate reductase, and BNF), suggesting that the nanoparticles stimulated N assimilation at this early stage. By contrast, in the late reproductive stage (R7), the same treatments were positioned closer to variables describing tissue Ni pools and farther from the BNF and nodulation vectors, indicating that the initial stimulation of N fixation was not maintained. In other words, although the plants continued to accumulate Ni, the biological benefits to N cycling diminished as the crop approached grain filling.

This transient “boost-then-decline” pattern may arise from several mechanisms, not mutually exclusive. Over time, nanoparticles can transform or dissolve, altering Ni speciation and bioavailability; internal Ni concentrations may reach levels that trigger feedback inhibition of key enzymes or delayed oxidative or phytotoxic effects may offset early gains. Similar early stimulation followed by later inhibition of microbial or plant enzymatic activity has been reported for other metal-based nanoparticles, including NiO and CuO.^110,111^

Nickel enhances nitrogen metabolism by increasing urease activity, regardless of the form of Ni used or how it is applied. As a key enzyme in N recycling, urease facilitates urea breakdown into ammonia, supporting overall N metabolism.^16^ Nitrate reductase activity improved significantly with nano Ni, particularly at the R2 stage, corresponding with peak nodulation.^69^ However, NR activity overall declined during the R5 stage compared with the previously stage analyzed (V6), as soybean enters the reproductive stage, and N demands shift toward seed filling.^112,113^ Additionally, macro-Ni application increased ureide synthesis at the R2 stage, crucial for N transport in soybean tissues.^114^ Despite elevated Ni levels in treated plants, no toxicity symptoms were observed, with concentrations remaining below thresholds for sensitive species. This highlights the safety and efficiency of nano-Ni fertilizers compared with conventional salts.

Accordingly, the controlled greenhouse conditions used in this study allowed for the isolation of nanoparticle–soil–plant interactions under well-defined temperature, moisture, and radiation regimes; however, they do not fully capture the complexity of field environments. Under field conditions, factors such as diurnal and seasonal temperature fluctuations, variable solar radiation, soil heterogeneity, and dynamic microbial activity may alter nanoparticle transformation, mobility, and Ni release kinetics. In particular, spatial variability in soil properties and root–microbe interactions may lead to heterogeneous Ni availability compared with the more uniform conditions of greenhouse experiments. Despite these limitations, greenhouse studies provide valuable mechanistic insight into early plant responses and nanoparticle behavior, serving as an important step toward understanding potential field-scale outcomes. Future field-based studies are therefore necessary to validate the extent to which the observed responses translate to agronomic conditions.

## 5 Implications and Outlook: Advancing Nutritional Genomics and Mechanistic Insights

In summary, this study evaluated the use of nickel nanoparticles (Ni­(OH)_2_) in soybean and found that, although seed + foliar applications produced a short-term stimulation of BNF, nitrogen metabolism, and seed Ni concentration at early reproductive stages, these benefits were not sustained through later growth. By grain filling, tissue Ni concentrations remained elevated, but key indicators of BNF and N metabolism had declined relative to their early-season levels, indicating that the initial advantages of the nanoparticle treatment were transient.

These results confirm the essential role of Ni as a cofactor in nitrogen metabolism but do not support the broad superiority of nano-Ni formulations over conventional sources. Instead, the findings highlight that the performance of Ni nanoparticles is strongly stage-dependent and may not provide consistent agronomic benefits across the entire soybean growth cycle. Additionally, when enzymatic activities and nitrogen-related metabolites measured by spectrophotometric assays are interpreted, it is important to consider potential analytical limitations associated with nanoparticle-based treatments. In this study, measurements were performed on clarified plant extracts rather than nanoparticle suspensions, which reduces the likelihood of direct optical interference during analysis. However, nanoparticle-specific assay controls and full spectral scans were not included. Therefore, while the observed trends are consistent with established physiological roles of Ni in plant nitrogen metabolism, subtle analytical interference cannot be completely excluded and should be addressed in future studies specifically designed to evaluate nanoparticle–assay interactions.

Beyond analytical considerations, the observed pattern of early stimulation followed by a decline in plant physiological responses suggests a complex interaction between Ni availability and plant demand. While the precise mechanisms underlying this temporal response remain uncertain, the strong relationship observed between Ni availability and soil pH in this study indicates that pH-mediated changes in Ni solubility may have contributed to this “boost–decline” pattern. Under acidic conditions, Ni nanoparticles are expected to exhibit increased dissolution and bioavailability, which may initially support nitrogen metabolism but subsequently lead to excessive Ni availability and potential toxicity, thereby impairing plant growth. Finally, the possible long-term ecological impacts and residual effects of Ni nanoparticles warrant careful evaluation. Studies that combine time-resolved nutrient monitoring with assessments of soil health and environmental fate are needed before the large-scale adoption of Ni-nanoparticle fertilizers. Overall, while Ni remains a key element for soybean nitrogen metabolism, our findings suggest that nano-Ni offers, at best, a temporary advantage rather than a consistent improvement in crop N-use efficiency.

## 6 Conclusions

This study shows that nickel nanoparticle (Ni­(OH)_2_) applications elicit a transient stimulation of soybean N metabolism and BNF rather than a sustained synergistic effect. Our hypothesis that nanosized particles would enhance Ni bioavailability relative to micrometric or conventional Ni sources was supported by the early reproductive stage (R2), when nano treatments promoted Ni uptake and were associated with higher urease and nitrate-reductase activity, as well as elevated levels of ammonia, nitrate, and ureides. These early enhancements coincided with improved BNF indicators, including nitrogenase activity and δ^15^N signatures.

However, as the crop progressed toward grain filling (R7), the initial gains diminished: tissue Ni concentrations remained elevated, yet BNF and related N-cycling parameters declined relative to earlier stages and some conventional Ni treatments. This stage-dependent “boost–then–decline” pattern suggests that while Ni nanoparticles can transiently enhance N assimilation and fixation, their benefits may not persist through late reproductive development.

These findings underscore the need to account for crop developmental stage when evaluating nanofertilizer performance. Future work should examine the mechanisms underlying the observed temporal decline, such as nanoparticle transformation, feedback inhibition, or delayed phytotoxicity, and assess long-term ecological and agronomic consequences. Only by understanding these dynamics can Ni­(OH)_2_-NP fertilizers be deployed sustainably and effectively in soybean and other cropping systems.

## Supplementary Material



## Notes

PCA analyses, supplementary histograms, additional figures, and tables (PDF)

## #

Department of Agronomy, Kansas State University, Throckmorton, 1712 Claflin Road, Manhattan, Kansas 66506, United States

## Abbreviations

biological nitrogen fixation: (BNF)
acetylene reduction assay: (ARA)
nitrate reductase: (NR)
nitrogenase;Ni-source based nanofertilizer: (Ni-NPs)

## References

[1] Singh P., Kumar R., Sabapathy S. N., Bawa A. S. (2008). Functional and Edible
Uses of Soy Protein Products. Compr. Rev. Food
Sci. Food Saf..

[2] Sinclair T. R., de Wit C. T. (1975). Photosynthate and
Nitrogen Requirements for Seed Production
by Various Crops. Science.

[3] Balboa G. R., Sadras V. O., Ciampitti I. A. (2018). Shifts
in Soybean Yield, Nutrient
Uptake, and Nutrient Stoichiometry: A Historical Synthesis-Analysis. Crop Sci..

[4] Salvagiotti F., Cassman K. G., Specht J. E., Walters D. T., Weiss A., Dobermann A. (2008). Nitrogen Uptake Fixation and Response
to Fertilizer
N in Soybeans: A Review. Field Crops Res..

[5] Hungria M., Araujo R. S., Silva Júnior E.
B., Zilli J. É. (2017). Inoculum Rate Effects on the Soybean Symbiosis in New or Old Fields
under Tropical Conditions. Agron. J..

[6] Cadisch G., Hairiah K., Giller K. E. (2000). Applicability of the Natural 15 N
Abundance Technique to Measure N 2 Fixation in Arachis Hypogaea Grown
on an Ultisol. Netherlands J. Agric. Sci..

[7] González-Guerrero M., Matthiadis A., Sáez Á., Long T. A. (2014). Fixating on Metals:
New Insights into the Role of Metals in Nodulation and Symbiotic Nitrogen
Fixation. Front. Plant Sci..

[8] Campo R. J., Araujo R. S., Hungria M. (2009). Nitrogen Fixation
with the Soybean
Crop in Brazil: Compatibility between Seed Treatment with Fungicides
and Bradyrhizobial Inoculants. Symbiosis.

[9] Jensen E. S., Hauggaard-Nielsen H. (2003). How Can Increased Use of Biological N2 Fixation in
Agriculture Benefit the Environment?. Plant
Soil.

[10] Alves B. J. R., Boddey R. M., Urquiaga S. (2003). The Success of BNF in Soybean in
Brazil. Entomol. Exp. Appl..

[11] Kebede E. (2021). Contribution
Utilization, and Improvement of Legumes-Driven Biological Nitrogen
Fixation in Agricultural Systems. Front. Sustain.
Food Syst.

[12] Fageria, N. K. ; Stone, L. F. Micronutrient Deficiency Problems in South America Micronutrient Deficiencies In Global Crop Production Springer 2008 245–266 10.1007/978-1-4020-6860-7_10

[13] Brown P. H., Welch R. M., Cary E. E. (1987). Nickel:
A Micronutrient Essential
for Higher Plants. Plant Physiol..

[14] Dixon N. E., Gazzola C., Blakeley R. L., Zerner B. (1975). Jack Bean Urease (EC
3.5.1.5). A Metalloenzyme. A Simple Biological Role for Nickel?. J. Am. Chem. Soc..

[15] Fabiano C. C., Tezotto T., Favarin J., Polacco J. C., Mazzafera P. (2015). Essentiality
of Nickel in Plants: A Role in Plant Stresses. Front. Plant Sci..

[16] Polacco J. C., Mazzafera P., Tezotto T. (2013). Plant Science Opinion – Nickel
and Urease in Plants: Still Many Knowledge Gaps. Plant Sci..

[17] Klucas R. V., Hanus F. J., Russell S. A., Evans H. J. (1983). Nickel: A Micronutrient
Element for Hydrogen-Dependent Growth of Rhizobium Japonicum and for
Expression of Urease Activity in Soybean Leaves. Proc. Natl. Acad. Sci. U. S. A..

[18] Lavres J., Franco G. C., de Sousa
Câmara G. M. (2016). Soybean Seed Treatment
with Nickel Improves Biological Nitrogen Fixation and Urease Activity. Front. Environ. Sci..

[19] Eskew D. L., Welch R. M., Cary E. E. (1983). Nickel:
An Essential Micronutrient
for Legumes and Possibly All Higher Plants. Science.

[20] Fernandez V., Brown P. H. (2013). From Plant Surface to Plant Metabolism:
The Uncertain
Fate of Foliar-Applied Nutrients. Front. Plant
Sci..

[21] Rodak B. W., Freitas D. S., Rossi M. L., Linhares F. S., Moro E., Campos C. N. S., Reis A. R., Guilherme L. R. G., Lavres J. (2024). A Study on Nickel Application Methods
for Optimizing
Soybean Growth. Sci. Rep..

[22] Farooq, M. ; Wahid, A. ; Basra, S. M. A. ; Khaliq, A. Rice Seed Invigoration: A Review. In Organic Farming, Pest Control and Remediation of Soil Pollutants; Springer 2009. 137–175. DOI: 10.1007/978-1-4020-9654-9

[23] Farooq M., Wahid A., Siddique K. H. M. (2012). Micronutrient Application through
Seed Treatments - a Review. J. Soil Sci. Plant
Nutr..

[24] Taylor A. G., Allen P. S., Bennett M. A., Bradford K. J., Burris J. S., Misra M. K. (1998). Seed Enhancements. Seed Sci.
Res..

[25] Basra S. M. A., Farooq M., Tabassam R., Ahmad N. (2005). Physiological
and Biochemical
Aspects of Pre-Sowing Seed Treatments in Fine Rice (Oryza Sativa L.). Seed Sci. Technol..

[26] Farooq M., Basra S. M. A., Khalid M., Tabassum R., Mahmood T. (2006). Nutrient Homeostasis,
Metabolism of Reserves, and Seedling Vigor as Affected by Seed Priming
in Coarse Rice. Can. J. Bot..

[27] Fageria N. K., Filho M. P. B., Moreira A., Guimarães C. M. (2009). Foliar
Fertilization of Crop Plants. J. Plant Nutr..

[28] De
Oliveira J. B., Lavres J., Kopittke P. M., Chaney R. L., Harris H. H., Erskine P. D., Howard D. L., Dos Reis A. R., Van Der Ent A. (2024). Unravelling the Fate of Foliar-Applied Nickel in Soybean:
A Comprehensive Investigation. Plant Soil.

[29] Kaya C., Higgs D. (2002). Response of Tomato
(Lycopersicon Esculentum L.) Cultivars to Foliar
Application of Zinc When Grown in Sand Culture at Low Zinc. Sci. Hortic..

[30] Freitas M. N., Guerra M. B. B., Adame A., Moraes T. F., Junior J. L., Pérez C. A., Abdala D. B., Cicero S. M. (2020). A First
Glance at
the Micro-ZnO Coating of Maize (*Zea Mays* L.) Seeds:
A Study of the Elemental Spatial Distribution and Zn Speciation Analysis. J. Anal. At. Spectrom..

[31] Kutman B. Y., Kutman U. B., Cakmak I. (2013). Nickel-Enriched
Seed and Externally
Supplied Nickel Improve Growth and Alleviate Foliar Urea Damage in
Soybean. Plant Soil.

[32] Migliavacca R. A., Gomes M. H. F., Ferraz-Almeida R., Almeida E. D., Lavres J., Carvalho H. W. P. D., Otto R. (2021). Comparison of Sources with Different
Solubilities for Mn Supply and Retranslocation along with Soybean
Development. J. Plant Nutr..

[33] Fernández, V. ; Sotiropoulos, T. ; Brown, P. . Foliar Fertilization: Scientific Principles And Field Pratices; IFA 2013.

[34] Niu J., Liu C., Huang M., Liu K., Yan D. (2021). Effects of Foliar Fertilization:
A Review of Current Status and Future Perspectives. J. Soil Sci. Plant Nutr..

[35] Bezerra
De Oliveira J., Rodrigues Marques J.
P., Rodak B. W., Galindo F. S., Carr N. F., Almeida E., Araki K., Gonçalves J. M., Rodrigues Dos Reis A., Van Der Ent A., Pereira De Carvalho H. W., Lavres J. (2022). Fate of Nickel in Soybean
Seeds Dressed with Different Forms of Nickel. Rhizosphere.

[36] Gomes M. H. F., Migliavacca R. A., Otto R., Carvalho H. W. P. (2020). Physicochemical
Characterization of Fertilizers Containing Concentrated Suspensions
of CuO, MnCO3 and ZnO. Sci. Agric..

[37] Santos E., Montanha G. S., Agostinho L. F., Polezi S., Marques J. P. R., De Carvalho H. W. P. (2023). Foliar
Calcium Absorption by Tomato
Plants: Comparing the Effects of Calcium Sources and Adjuvant Usage. Plants.

[38] Faizan M., Singh A., Eren A., Sultan H., Sharma M., Djalovic I., Trivan G. (2024). Small Molecule Big Impacts: Nano-Nutrients
for Sustainable Agriculture and Food Security. J. Plant Physiol..

[39] Kondak S., Kondak D., Kabadayi O., Erdei L., Rónavári A., Kónya Z., Galbács G., Kolbert Z. (2024). Current insights into
the green synthesis, in planta characterization and phytoeffects of
nickel nanoparticles and their agricultural implications. Environmental Research.

[40] Thangavelu R. M., Silva W. L. D., Zuverza-Mena N., Dimkpa C. O., White J. C. (2024). Nano-Sized
Metal Oxide Fertilizers for Sustainable Agriculture: Balancing Benefits,
Risks, and Risk Management Strategies. Nanoscale.

[41] Wei L., Liu J., Jiang G. (2024). Nanoparticle-Specific
Transformations Dictate Nanoparticle
Effects Associated with Plants and Implications for Nanotechnology
Use in Agriculture. Nat. Commun..

[42] Thirumalaisamy R., Nimithap S., Harini S., Rajiniganth R., Haritha S., Ameer K., Vadivel P., Selvankumar T. (2025). Potential
Application of Nanoemulsion in Agriculture: A Current Perspective. IJASE.

[43] Kah M., Kookana R. S., Gogos A., Bucheli T. D. (2018). A Critical Evaluation
of Nanopesticides and Nanofertilizers against Their Conventional Analogues. Nat. Nanotechnol..

[44] White J. C., Gardea-Torresdey J. (2018). Achieving
Food Security through the Very Small. Nat. Nanotechnol..

[45] Santás-Miguel V., Arias-Estévez M., Rodríguez-Seijo A., Arenas-Lago D. (2023). Use of Metal
Nanoparticles in Agriculture. A Review on the Effects on Plant Germination. Environ. Pollut..

[46] Santos E., Montanha G. S., Gomes M. H. F., Duran N. M., Corrêa C. G., Romeu S. L. Z., Pereira A. E. S., Oliveira J. L., Almeida E., Pérez-de-Luque A. (2022). Are Nanomaterials Leading
to More Efficient Agriculture? Outputs from 2009 to 2022 Research
Metadata Analysis. Environ. Sci.: Nano.

[47] Ahmed D. F., Isawi H., Badway N. A., Elbayaa A. A., Shawky H. (2021). Graphene Oxide
Incorporated Cellulose Triacetate/Cellulose Acetate Nanocomposite
Membranes for Forward Osmosis Desalination. Arabian J. Chem..

[48] Nongbet A., Mishra A. K., Mohanta Y. K., Mahanta S., Ray M. K., Khan M., Baek K.-H., Chakrabartty I. (2022). Nanofertilizers:
A Smart and Sustainable Attribute to Modern Agriculture. Plants.

[49] Ghormade V., Deshpande M. V., Paknikar K. M. (2011). Perspectives for Nano-Biotechnology
Enabled Protection and Nutrition of Plants. Biotechnol. Adv..

[50] Liu R., Lal R. (2015). Potentials of Engineered
Nanoparticles as Fertilizers for Increasing
Agronomic Productions. Sci. Total Environ.

[51] da
Cruz T. N. M., Savassa S. M., Montanha G. S., Ishida J. K., de Almeida E., Tsai S. M., Lavres J., Pereira de Carvalho H. W. (2019). A New Glance on Root-to-Shoot in
Vivo Zinc Transport and Time-Dependent Physiological Effects of ZnSO4
and ZnO Nanoparticles on Plants. Sci. Rep..

[52] Subramanian, K. S. ; Manikandan, A. ; Thirunavukkarasu, M. ; Rahale, C. S. Nano-Fertilizers for Balanced Crop Nutrition Nanotechnologies In Food And Agriculture Springer 2015 69–80 10.1007/978-3-319-14024-7

[53] Duran N. M., Savassa S. M., Lima R. G. D., De Almeida E., Linhares F. S., Van Gestel C. A. M., Pereira De Carvalho H. W. (2017). X-Ray Spectroscopy
Uncovering the Effects of Cu Based Nanoparticle Concentration and
Structure on Phaseolus Vulgaris Germination and Seedling Development. J. Agric. Food Chem..

[54] Sun H., Li Z., Wen J., Zhou Q., Gong Y., Zhao X., Mao H. (2023). Co-Exposure
of Maize to Polyethylene Microplastics and ZnO Nanoparticles:
Impact on Growth, Fate, and Interaction. Sci.
Total Environ..

[55] Rodríguez-Seijo A., Soares C., Ribeiro S., Amil B. F., Patinha C., Cachada A., Fidalgo F., Pereira R. (2022). Nano-Fe2O3 as a Tool
to Restore Plant Growth in Contaminated Soils – Assessment
of Potentially Toxic Elements (Bio)­Availability and Redox Homeostasis
in *Hordeum Vulgare* L. J. Hazard.
Mater..

[56] Dilnawaz F., Misra A. N., Apostolova E. (2023). Involvement
of Nanoparticles in Mitigating
Plant’s Abiotic Stress. Plant Stress.

[57] Montanha G. S., Rodrigues E. S., Marques J. P. R., de Almeida E., Colzato M., Pereira
de Carvalho H.
W. (2020). Zinc Nanocoated Seeds:
An Alternative to Boost Soybean Seed Germination and Seedling Development. SN Appl. Sci..

[58] Savassa S. M., Duran N. M., Rodrigues E. S., De Almeida E., Van Gestel C. A. M., Bompadre T. F. V., De
Carvalho H. W. P. (2018). Effects
of ZnO Nanoparticles on Phaseolus Vulgaris Germination and Seedling
Development Determined by X-Ray Spectroscopy. ACS Appl. Nano Mater..

[59] Bakht, B. K. ; Iftikhar, M. ; Gul, I. ; Ali, M. A. ; Shah, G. M. ; Arshad, M. Chapter 9 - Effect of Nanoparticles on Crop Growth. In Nanomaterials for Soil Remediation; Elsevier, 2021; pp. 183–201. DOI: 10.1016/B978-0-12-822891-3.00009-8.

[60] Zhou P., Jiang Y., Adeel M., Shakoor N., Zhao W., Liu Y., Li Y., Li M., Azeem I., Rui Y., Tan Z., White J. C., Guo Z., Lynch I., Zhang P. (2023). Nickel Oxide
Nanoparticles Improve Soybean Yield and Enhance Nitrogen Assimilation. Environ. Sci. Technol..

[61] Embrapa. Sistema Brasileiro de classificação de Solos; Embrapa, 2018.

[62] Soil Survey Staff. Keys To Soil Taxonomy; USDA, 2022

[63] Raij, B. V. ; Andrade, J. C. ; Cantarella, H. ; Quaggio, J. A. . Análise Química Para Avaliação Da Fertilidade de Solos Tropicais; Instituto Agronômico. 2001

[64] Hatfield J. L., Egli D. B., Leggett J. E., Peaslee D. E. (1974). Effect of Applied
Nitrogen on the Nodulation and Early Growth of Soybeans (Glycine Max
(L.) MERR.) 1. Agron. J..

[65] Pierozan C., Favarin J. L., de Almeida R. E. M., Maciel de Oliveira S., Lago B. C., Trivelin P. C. O. (2015). Uptake
and Allocation of Nitrogen
Applied at Low Rates to Soybean Leaves. Plant
Soil.

[66] Rocha M. A., Winnischofer H., Araki K., Anaissi F. J., Toma H. E. (2011). A New Insight
on the Preparation of Stabilized Alpha-Nickel Hydroxide Nanoparticles. J. Nanosci. Nanotechnol..

[67] Nunes C. V., Danczuk M., Bortoti A. A., Gonçalves J. M., Araki K., Anaissi F. J. (2015). Unexpected Effect
of Drying Method
on the Microstructure and Electrocatalytic Properties of Bentonite/Alpha-Nickel
Hydroxide Nanocomposite. J. Power Sources.

[68] Martins P. R., Ferreira L. M. C., Araki K., Angnes L. (2014). Influence of Cobalt
Content on Nanostructured Alpha-Phase-Nickel Hydroxide Modified Electrodes
for Electrocatalytic Oxidation of Isoniazid. Sens. Actuators, B.

[69] Câmara G. M. (2014). Fixação
Biológica de Nitrogênio Em Soja. Informações Agronômicas.

[70] Boddey R. M., Polidoro J. C., Resende A. S., Alves B. J. R., Urquiaga S. (2001). Use of the
15N Natural Abundance Technique for the Quantification of the Contribution
of N2 Fixation to Sugar Cane and Other Grasses. Aust. J. Plant Physiol..

[71] Brito M. D. M. P., Muraoka T., Silva E. (2009). MARCHA DE
ABSORÇÃO
DO NITROGÊNIO DO SOLO, DO FERTILIZANTE E DA FIXAÇÃO
SIMBIÓTICA EM FEIJÃO-CAUPI (Vigna Unguiculata (L.) WALP.)
E FEIJÃO-COMUM (Phaseolus Vulgaris L.) DETERMINADA COM USO
DE 15N. R. Bras. Ci. Solo..

[72] Peoples, M.B. ; Faizah, A.W. ; Rerkasem, B. ; Herridge, D.F. Methods for Evaluating Nitrogen Fixation by Nodulated Legumes in the Field Australian Centre For International Agricultural Research 1989

[73] Urquiaga S., Xavier R. P., Morais R. F. D., Batista R. B., Schultz N., Leite J. M., Maia
e Sá J., Barbosa K. P., de Resende A. S., Alves B. J. R. (2012). Evidence from Field Nitrogen Balance and 15
N Natural Abundance Data for the Contribution of Biological N 2 Fixation
to Brazilian Sugarcane Varieties. Plant Soil.

[74] Fehr W. R., Caviness C. E., Burmood D. T., Pennington J. S. (1971). Stage of
Development Descriptions for Soybeans, Glycine Max (L.) Merrill 1. Crop Sci..

[75] Barcelos J. P. D. Q., Osório C. R. W. D. S., Leal A. J. F., Alves C. Z., Santos E. F., Reis H. P. G., dos Reis A. R. (2017). Effects of Foliar
Nickel (Ni) Application on Mineral Nutrition Status, Urease Activity
and Physiological Quality of Soybean Seeds. Aust. J. Crop Sci..

[76] Lavres J., Silveira Rabêlo F. H., Capaldi F. R., dos Reis A. R., Rosssi M. L., Franco M. R., Azevedo R. A., Abreu-Junior C. H., de Lima Nogueira N. (2019). Investigation into the Relationship
among Cd Bioaccumulation,
Nutrient Composition, Ultrastructural Changes and Antioxidative Metabolism
in Lettuce Genotypes under Cd Stress. Ecotoxicol.
Environ. Saf..

[77] US EPA. U.S. EPA Method 3051A: Microwave Assisted Acid Digestion of Sediments, Sludges, and Oils https://www.epa.gov/esam/us-epa-method-3051a-microwave-assisted-acid-digestion-sediments-sludges-and-oils (Accessed 02January 2025).

[78] Shearer G., Kohl D. H. (1988). Natural15N Abundance as a Method of Estimating the
Contribution of Biologically Fixed Nitrogen to N2-Fixing Systems:
Potential for Non-Legumes. Plant Soil.

[79] Trivelin, P. C. O. ; Coleti, J. T. ; Lara Cabezas, W. A. R. Efeito Residual Na Soqueira de Cana-de-Açúcar Do Nitrogênio Da Uréia Aplicada Por via Foliar Na Cana-Planta Anais 1985

[80] Guimarães A. P., Morais R. F. D., Urquiaga S., Boddey R. M., Alves B., Alves R. (2008). Bradyrhizobium STRAIN AND THE 15 N NATURAL ABUNDANCE QUANTIFICATION
OF BIOLOGICAL N 2 FIXATION IN SOYBEAN. Scientia
Agricola.

[81] Barrie A., Brookes S. T., Prosser S. J., Debney S. (1995). High Productivity Analysis
of15N and13C in Soil/Plant Research. Fert. Res..

[82] Hogan M. E., Swift I. E., Done J. (1983). Urease Assay and Ammonia Release
from Leaf Tissues. Phytochemistry.

[83] Mulder E. G., Boxma R., Van Veen W. L. (1959). The Effect
of Molybdenum and Nitrogen
Deficiencies on Nitrate Reduction in Plant Tissues. Plant Soil.

[84] McCullough H. (1967). The Determination
of Ammonia in Whole Blood by a Direct Colorimetric Method. Clin. Chim. Acta.

[85] Vogels G. D., Van Der Drift C. (1970). Differential
Analyses of Glyoxylate Derivatives. Anal. Biochem..

[86] Bates D., Mächler M., Bolker B., Walker S. (2015). Fitting Linear Mixed-Effects
Models Using **Lme4**. J. Stat. Soft..

[87] Wickham, H. ; François, R. ; Henry, L. ; Müller, K. ; Vaughan, D. ; Dplyr: A Grammar of Data Manipulation, 2023. https://cran.r-project.org/web/packages/dplyr/index.html (Accessed 02 January 2025).

[88] Wickham, H. ; Vaughan, D. ; Girlich, M. ; Ushey, K. ; Tidyr: Tidy Messy Data, 2024. https://cran.r-project.org/web/packages/tidyr/index.html (Accessed January 2025).

[89] () Lenth, R. V. ; Banfai, B. ; Bolker, B. ; Buerkner, P. ; Giné-Vázquez, I. ; Herve, M. ; Jung, M. ; Love, J. ; Miguez, F. ; Piaskowski, J. ; Riebl, H. ; Singmann, H. Emmeans: Estimated Marginal Means, Aka Least-Squares Means, 2024. https://cran.r-project.org/web/packages/emmeans/index.html (accessed 2025–01–02).

[90] () Kassambara, A. ; Mundt, F. Factoextra: Extract and Visualize the Results of Multivariate Data Analyses, 2020. https://cran.r-project.org/web/packages/factoextra/index.html (Accessed 07 January 2025).

[91] () Kolde, R. Pheatmap: Pretty Heatmaps, 2019. https://cran.r-project.org/web/packages/pheatmap/index.html (Accessed 07January 2025).

[92] Wickham, H. ; Chang, W. ; Henry, L. ; Pedersen, T. L. ; Takahashi, K. ; Wilke, C. ; Woo, K. ; Yutani, H. ; Dunnington, D. ; Brand, T. V. D. ; Posit; PBC. Ggplot2: Create Elegant Data Visualisations Using the Grammar of Graphics, 2024. https://cran.r-project.org/web/packages/ggplot2/index.html (Accessed 02 January 2025).

[93] Faisal M., Saquib Q., Alatar A. A., Al-Khedhairy A. A., Hegazy A. K., Musarrat J. (2013). Phytotoxic Hazards
of NiO-Nanoparticles
in Tomato: A Study on Mechanism of Cell Death. J. Hazard. Mater..

[94] Saleh A. M., Hassan Y. M., Selim S., AbdElgawad H. (2019). NiO-Nanoparticles
Induce Reduced Phytotoxic Hazards in Wheat (Triticum Aestivum L.)
Grown under Future Climate CO2. Chemosphere.

[95] Pandey V. K., Gopal R. (2010). Nickel Toxicity Effects on Growth and Metabolism of Eggplant. Int. J. Veg. Sci..

[96] Reis A. R. D., De Queiroz Barcelos J. P., De Souza Osório C. R. W., Santos E. F., Lisboa L. A. M., Santini J. M. K., Dos
Santos M. J. D., Furlani Junior E., Campos M., De Figueiredo P. A. M., Lavres J., Gratão P. L. (2017). A Glimpse into the Physiological,
Biochemical and Nutritional Status of Soybean Plants under Ni-Stress
Conditions. Environ. Exp. Bot..

[97] Uruç
Parlak K. (2016). Effect of Nickel on Growth and Biochemical Characteristics of Wheat
(Triticum Aestivum L.) Seedlings. NJAS - NJAS
Wageningen J. Life Sci..

[98] Martins P. R., Toma S. H., Nakamura M., Toma H. E., Araki K. (2013). Thermodynamic
Stabilization of Nanostructured Alpha-Ni1–xCox­(OH)­2 for High
Efficiency Batteries and Devices. RSC Adv..

[99] Watson J.-L., Fang T., Dimkpa C. O., Britt D. W., McLean J. E., Jacobson A., Anderson A. J. (2015). The Phytotoxicity
of ZnO Nanoparticles
on Wheat Varies with Soil Properties. BioMetals.

[100] Cyprichová V., Urík M., Csibriová S., Kolenčík M., Bujdoš M., Matúš P., Šebesta M. (2025). Interaction
of Zinc Oxide Nanoparticles
with Soil Colloidal Suspensions. Chemosphere.

[101] Tang C., Unkovich M. J., Bowden J. W. (1999). Factors
Affecting
Soil Acidification under Legumes. III. Acid Production by N_2_ -fixing Legumes as Influenced by Nitrate Supply. New Phytol..

[102] Cornelis G., Hund-Rinke K., Kuhlbusch T., Van Den Brink N., Nickel C. (2014). Fate and Bioavailability
of Engineered
Nanoparticles in Soils: A Review. Crit. Rev.
Environ. Sci. Technol..

[103] Kutman B. Y., Kutman U. B., Cakmak I. (2014). Effects of
Seed Nickel
Reserves or Externally Supplied Nickel on the Growth, Nitrogenmetabolites
and Nitrogen Use Efficiency of Urea- or Nitrate-Fed Soybean. Plant Soil.

[104] Yusuf M., Fariduddin Q., Hayat S., Ahmad A. (2011). Nickel: An
Overview of Uptake, Essentiality and Toxicity in Plants. Bull. Environ. Contam. Toxicol..

[105] Arora P. K., Tripathi S., Omar R. A., Chauhan P., Sinhal V. K., Singh A., Srivastava A., Garg S. K., Singh V. P. (2024). Next-Generation Fertilizers: The
Impact of Bionanofertilizers on Sustainable Agriculture. Microb. Cell Fact..

[106] Babaahmadifooladi M., Jacxsens L., Meulenaer B. D., Laing G. D. (2020). Nickel in Foods Sampled on the Belgian Market: Identification
of Potential Contamination Sources. Food Addit.
Contam., Part A.

[107] Rossi L., Fedenia L. N., Sharifan H., Ma X., Lombardini L. (2019). Effects of Foliar Application of Zinc Sulfate and Zinc
Nanoparticles in Coffee (Coffea Arabica L.) Plants. Plant Physiol. Biochem..

[108] Freitas D., Wurr Rodak B., Rodrigues dos Reis A., de Barros Reis F., Soares de Carvalho T., Schulze J., Carbone
Carneiro M. A., Guimarães Guilherme L. R. (2018). Hidden Nickel Deficiency?
Nickel Fertilization via Soil Improves Nitrogen Metabolism and Grain
Yield in Soybean Genotypes. Front. Plant Sci..

[109] Freitas D. S., Rodak B. W., Carneiro M. A. C., Guilherme L. R. G. (2019). How
Does Ni Fertilization Affect a Responsive Soybean Genotype? A Dose
Study. Plant Soil.

[110] Kolesnikov S., Timoshenko A., Minnikova T., Tsepina N., Kazeev K., Akimenko Y., Zhadobin A., Shuvaeva V., Rajput V. D., Mandzhieva S., Sushkova S., Minkina T., Dudnikova T., Mazarji M., Alamri S., Siddiqui M. H., Singh R. K. (2021). Impact
of Metal-Based Nanoparticles on Cambisol Microbial Functionality,
Enzyme Activity, and Plant Growth. Plants.

[111] Shah G. A., Ahmed J., Iqbal Z., Hassan F.-., Rashid M. I. (2021). Toxicity of NiO Nanoparticles to
Soil Nutrient Availability
and Herbage N Uptake from Poultry Manure. Sci.
Rep..

[112] Karwat H., Sparke M.-A., Rasche F., Arango J., Nuñez J., Rao I., Moreta D., Cadisch G. (2019). Nitrate Reductase
Activity in Leaves as a Plant Physiological Indicator of in Vivo Biological
Nitrification Inhibition by Brachiaria Humidicola. Plant Physiol. Biochem..

[113] Nicholas J. C., Harper J. E., Hageman R. H. (1976). Nitrate Reductase
Activity in Soybeans (Glycine Max [L.] Merr.). Plant Physiol..

[114] Todd C. D., Tipton P. A., Blevins D. G., Piedras P., Pineda M., Polacco J. C. (2006). Update on Ureide Degradation in Legumes. J. Exp. Bot..

