# Supplementary material for: Nickel Supplementation for Enhanced Soybean Growth: A Micrometric-to-Nanometric Investigation of Biological Nitrogen Fixation and Metabolism
Source: ACS Omega. 2026 Apr 24;11(17):25297–316. doi: 10.1021/acsomega.5c10937 (PMC13150659; doi:10.1021/acsomega.5c10937)
Supplement: Supplementary file 1 [file ao5c10937_si_001.pdf]

ELECTRONIC SUPPLEMENTARY INFORMATION

Nickel Supplementation for Enhanced Soybean Growth: A Micrometric-to-Nanometric Investigation of Biological Nitrogen Fixation and Metabolism

Jessica Bezerra de Oliveira[11*], Antony van der Ent[2], Fernanda Viginotti Alves[1], Bruna Wurr Rodak[1], Nikolas de Souza Mateus[1], Nandhara Angelica Carvalho Mendes[3], Josué Martins Gonçalves[3], Koiti Araki[4], Hudson Wallace Pereira de Carvalho[1], André Rodrigues dos Reis[3], Fernando Shintate Galindo[5], José Lavres[1]

[1]*Universidade de São Paulo, Centro de Energia Nuclear na Agricultura, Brazil.*

[2] *Wageningen University & Research, Laboratory of Genetics, Wageningen, The Netherlands*

[3]*Sao Paulo State University (UNESP), Tupã, Brazil.*

[4]*University of São Paulo, Institute of Chemistry, Department of Fundamental Chemistry, São Paulo, São Paulo, 05508-000, Brazil.*

[5]*Sao Paulo State University (UNESP), Dracena, Brazil.*

*Corresponding author. E-mail address: jbezerra@ksu.edu

Department, 2734

Phone number: +1 785 410-5169

Date of submission: December 04, 2025

Word count: 16249

Figure: 2

---

[1] Current address: Kansas State University, 1712 Claflin Road, Throckmorton, Agronomy

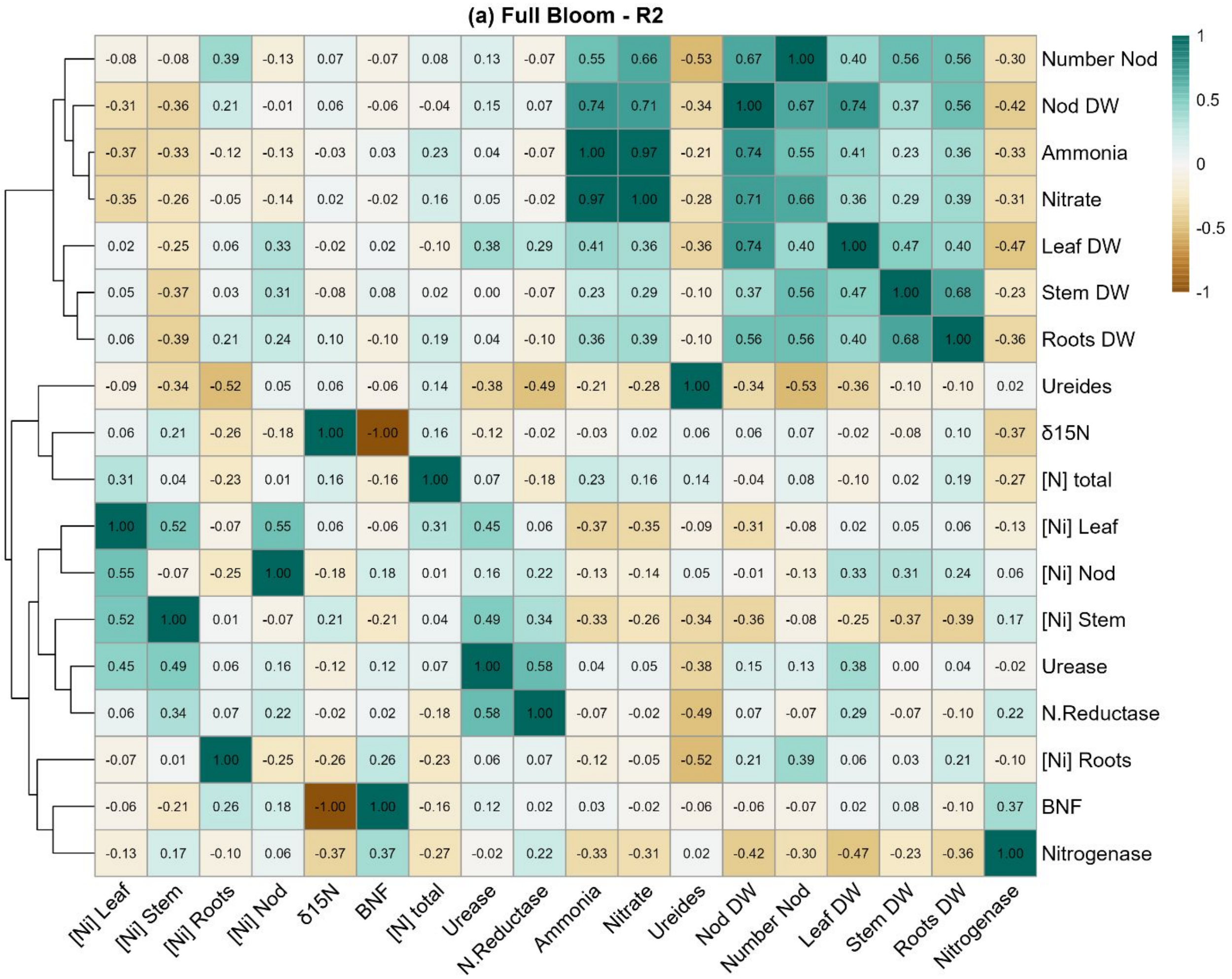
(a) Full Bloom - R2
Number Nod -0.08 -0.08 0.39 -0.13 0.07 -0.07 0.08 0.13 -0.07 0.55 0.66 -0.53 0.67 1.00 0.40 0.56 0.56 -0.30
Nod DW -0.31 -0.36 0.21 -0.01 0.06 -0.06 -0.04 0.15 0.07 0.74 0.71 -0.34 1.00 0.67 0.74 0.37 0.56 -0.42
Ammonia -0.37 -0.33 -0.12 -0.13 -0.03 0.03 0.23 0.04 -0.07 1.00 0.97 -0.21 0.74 0.55 0.41 0.23 0.36 -0.33
Nitrate -0.35 -0.26 -0.05 -0.14 0.02 -0.02 0.16 0.05 -0.02 0.97 1.00 -0.28 0.71 0.66 0.36 0.29 0.39 -0.31
Leaf DW 0.02 -0.25 0.06 0.33 -0.02 0.02 -0.10 0.38 0.29 0.41 0.36 -0.36 0.74 0.40 1.00 0.47 0.40 -0.47
Stem DW 0.05 -0.37 0.03 0.31 -0.08 0.08 0.02 0.00 -0.07 0.23 0.29 -0.10 0.37 0.56 0.47 1.00 0.68 -0.23
Roots DW 0.06 -0.39 0.21 0.24 0.10 -0.10 0.19 0.04 -0.10 0.36 0.39 -0.10 0.56 0.56 0.40 0.68 1.00 -0.36
Ureides -0.09 -0.34 -0.52 0.05 0.06 -0.06 0.14 -0.38 -0.49 -0.21 -0.28 1.00 -0.34 -0.53 -0.36 -0.10 -0.10 0.02
δ15N 0.06 0.21 -0.26 -0.18 1.00 -1.00 0.16 -0.12 -0.02 -0.03 0.02 0.06 0.06 0.07 -0.02 -0.08 0.10 -0.37
[N] total 0.31 0.04 -0.23 0.01 0.16 -0.16 1.00 0.07 -0.18 0.23 0.16 0.14 -0.04 0.08 -0.10 0.02 0.19 -0.27
[Ni] Leaf 1.00 0.52 -0.07 0.55 0.06 -0.06 0.31 0.45 0.06 -0.37 -0.35 -0.09 -0.31 -0.08 0.02 0.05 0.06 -0.13
[Ni] Nod 0.55 -0.07 -0.25 1.00 -0.18 0.18 0.01 0.16 0.22 -0.13 -0.14 0.05 -0.01 -0.13 0.33 0.31 0.24 0.06
[Ni] Stem 0.52 1.00 0.01 -0.07 0.21 -0.21 0.04 0.49 0.34 -0.33 -0.26 -0.34 -0.36 -0.08 -0.25 -0.37 -0.39 0.17
Urease 0.45 0.49 0.06 0.16 -0.12 0.12 0.07 1.00 0.58 0.04 0.05 -0.38 0.15 0.13 0.38 0.00 0.04 -0.02
N.Reductase 0.06 0.34 0.07 0.22 -0.02 0.02 -0.18 0.58 1.00 -0.07 -0.02 -0.49 0.07 -0.07 0.29 -0.07 -0.10 0.22
[Ni] Roots -0.07 0.01 1.00 -0.25 -0.26 0.26 -0.23 0.06 0.07 -0.12 -0.05 -0.52 0.21 0.39 0.06 0.03 0.21 -0.10
BNF -0.06 -0.21 0.26 0.18 -1.00 1.00 -0.16 0.12 0.02 0.03 -0.02 -0.06 -0.06 -0.07 0.02 0.08 -0.10 0.37
Nitrogenase -0.13 0.17 -0.10 0.06 -0.37 0.37 -0.27 -0.02 0.22 -0.33 -0.31 0.02 -0.42 -0.30 -0.47 -0.23 -0.36 1.00
[Ni] Leaf
[Ni] Stem
[Ni] Roots
[Ni] Nod
δ15N
BNF
[N] total
Urease
N.Reductase
Ammonia
Nitrate
Ureides
Nod DW
Number Nod
Leaf DW
Stem DW
Roots DW
Nitrogenase
1
0.5
0
-0.5
-1

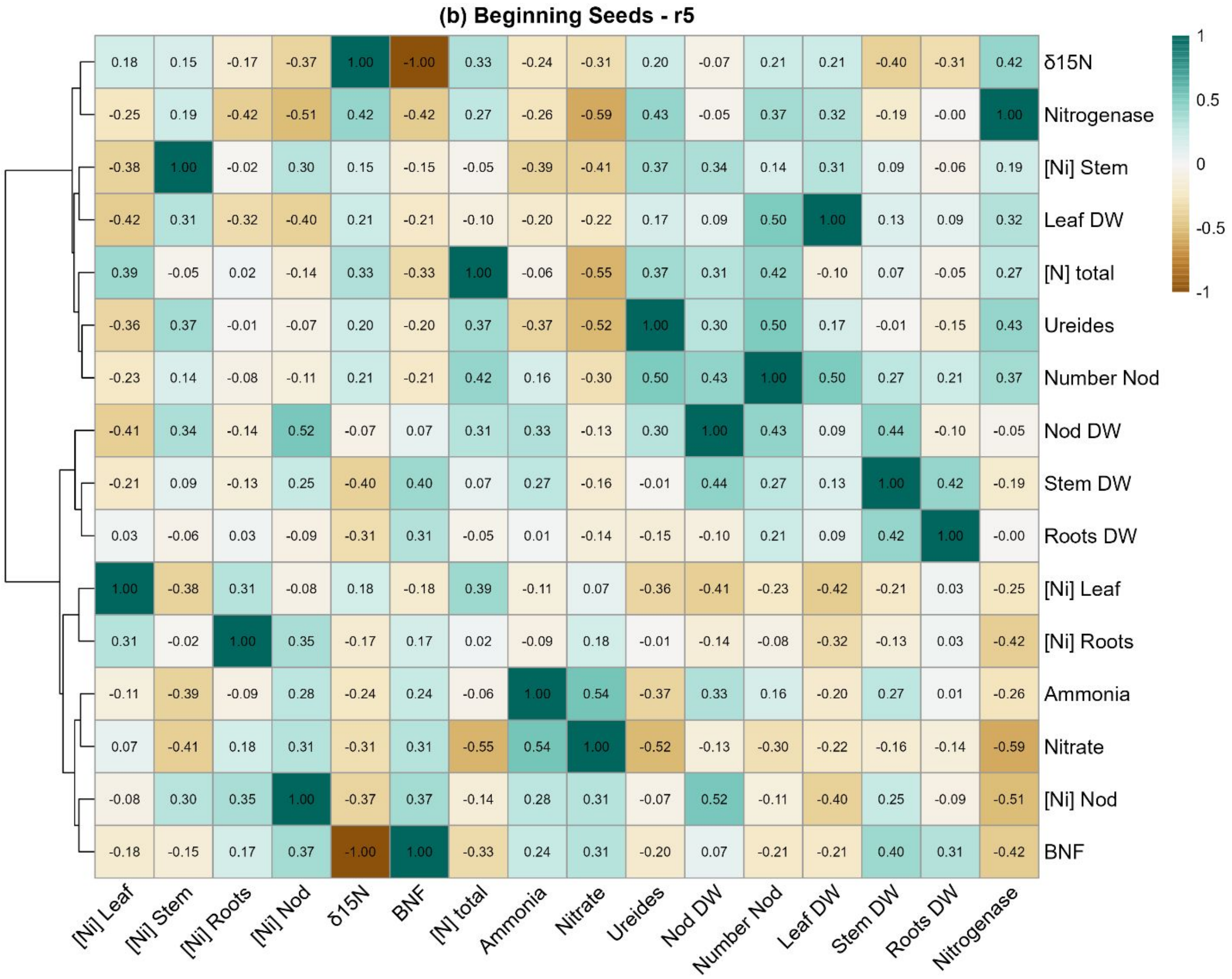
(b) Beginning Seeds - r5
δ15N
Nitrogenase
[Ni] Stem
Leaf DW
[N] total
Ureides
Number Nod
Nod DW
Stem DW
Roots DW
[Ni] Leaf
[Ni] Roots
Ammonia
Nitrate
[Ni] Nod
BNF
[Ni] Leaf
[Ni] Stem
[Ni] Roots
[Ni] Nod
δ15N
BNF
[N] total
Ammonia
Nitrate
Ureides
Nod DW
Number Nod
Leaf DW
Stem DW
Roots DW
Nitrogenase
1
0.5
0
-0.5
-1

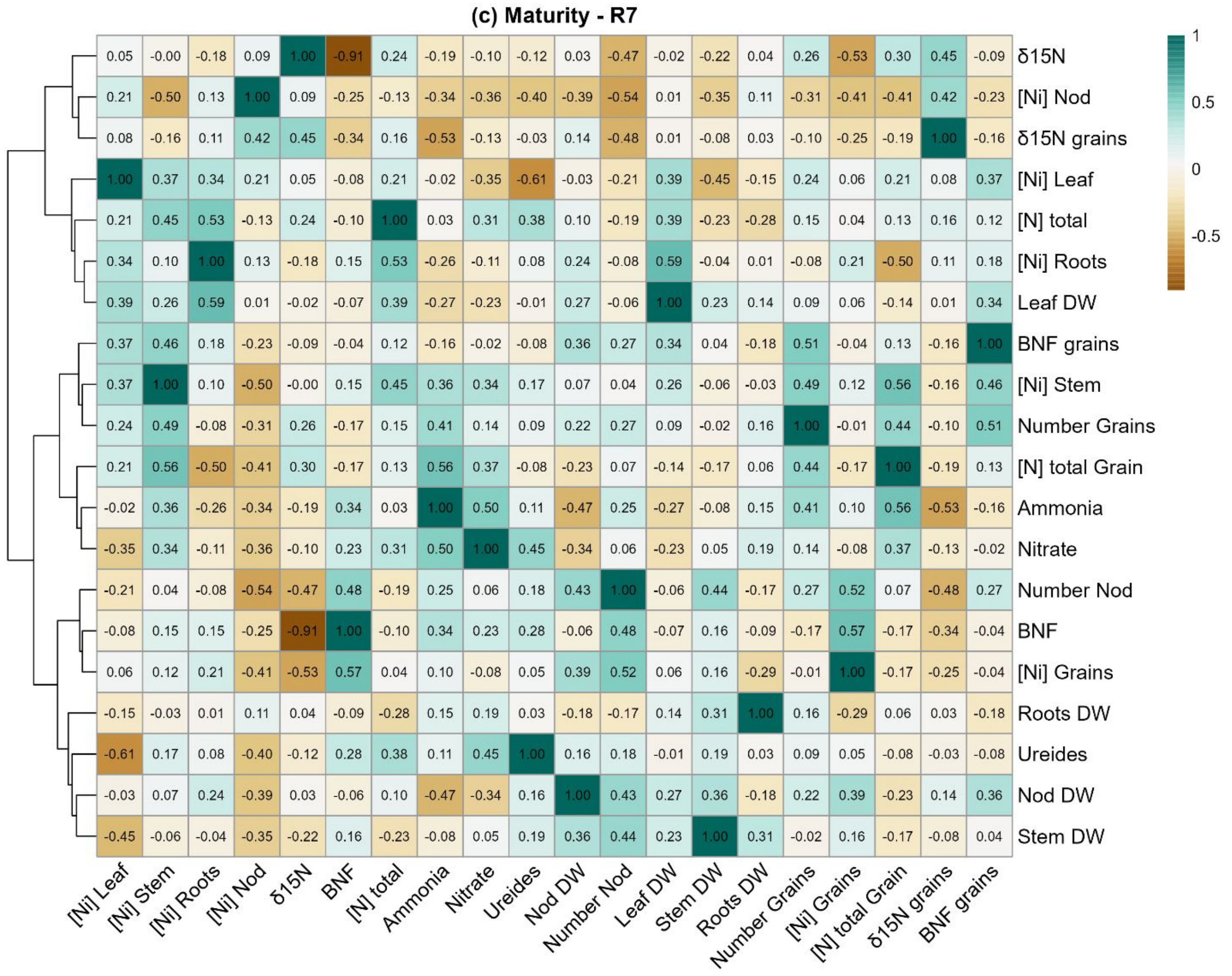

Figure S1. Pearson correlations and hierarchical cluster analyses of soybean phenological stages (a) Flowering (R2); (b) Beginning seeds (R5); and (c) maturity (R7).

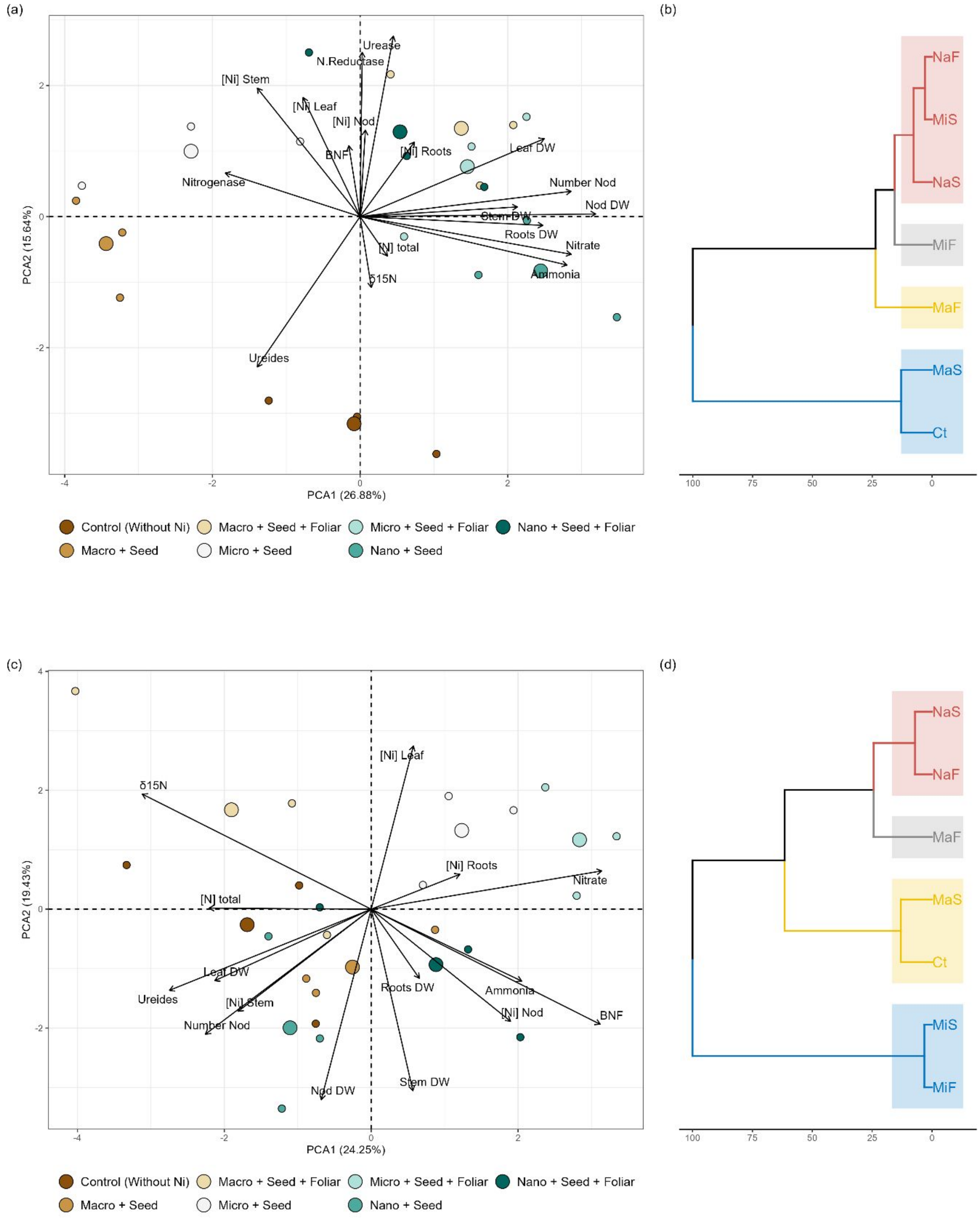
(a)
(b)
Urease
N.Reductase
[Ni] Stem
[Ni] Leaf
[Ni] Nod
BNF
[Ni] Roots
Leaf DW
Nitrogenase
Number Nod
Nod DW
Stem DW
Roots DW
Nitrate
[N] total
Ammonia
δ15N
Ureides
PCA1 (26.88%)
PCA2 (15.64%)
NaF
MiS
NaS
MiF
MaF
MaS
Ct
100
75
50
25
0
Control (Without Ni)
Macro + Seed + Foliar
Micro + Seed + Foliar
Nano + Seed + Foliar
Macro + Seed
Micro + Seed
Nano + Seed
(c)
(d)
[Ni] Leaf
δ15N
[Ni] Roots
Nitrate
[N] total
Leaf DW
Ureides
[Ni] Stem
Number Nod
Roots DW
Ammonia
[Ni] Nod
BNF
Stem DW
Nod DW
PCA1 (24.25%)
PCA2 (19.43%)
NaS
NaF
MaF
MaS
Ct
MiS
MiF
100
75
50
25
0
Control (Without Ni)
Macro + Seed + Foliar
Micro + Seed + Foliar
Nano + Seed + Foliar
Macro + Seed
Micro + Seed
Nano + Seed

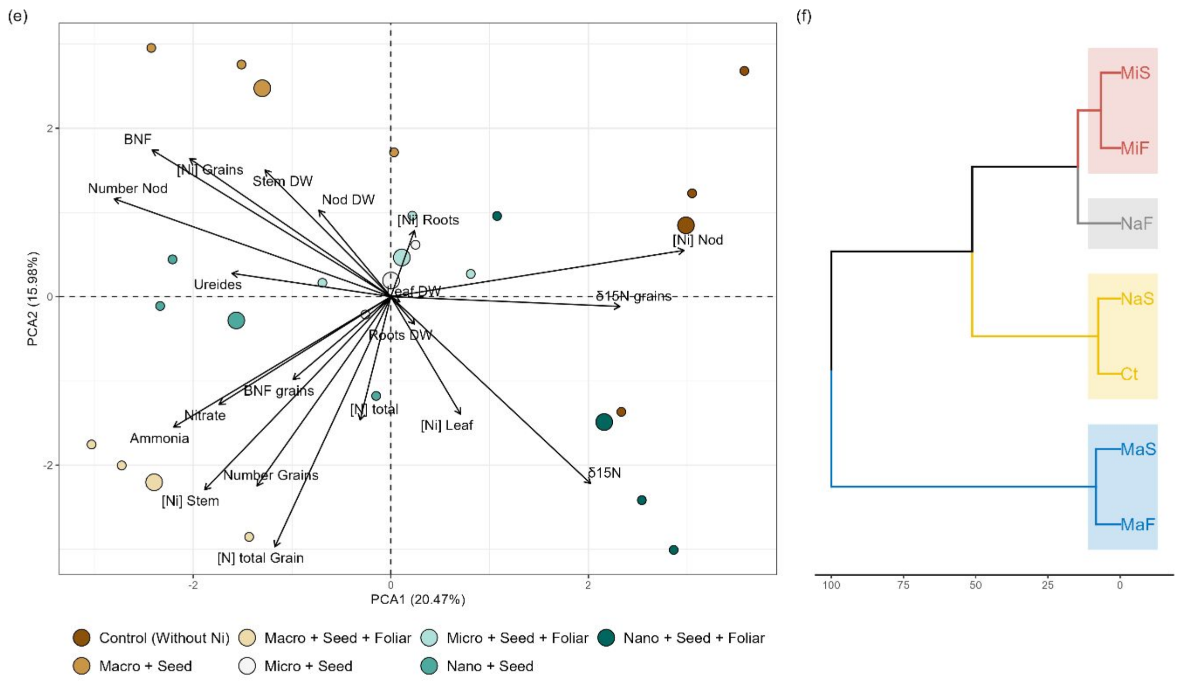

Figure S2. Principal Component Analysis (PCA) (a, c, e) and hierarchical cluster analysis (b, d, f) of soybean phenological stages: (a-b) Full bloom (R2), (c-d) Beginning seeds (R5), and (e-f) Maturity (R7). Treatment abbreviations are as follows: NaF = Nano + Seed + Foliar, MiS = Micro + Seed, NaS = Nano + Seed, MiF = Micro + Seed + Foliar, MaF = Macro + Seed + Foliar, MaS = Macro + Seed, Ct = Control (Without Ni).
